# Supplementary material for: Synthesis and Inhibitory Activity of Machaeridiol-Based Novel Anti-MRSA and Anti-VRE Compounds and Their Profiling for Cancer-Related Signaling Pathways
Source: Molecules. 2022 Oct 5;27(19):6604. doi: 10.3390/molecules27196604 (PMC9570708; doi:10.3390/molecules27196604)
Supplement: Supplementary file 1 [file molecules-27-06604-s001.zip › molecules-1932320-supplementary.pdf]

Supporting Information

Synthesis and Inhibitory Activity of Machaeridiol- based Novel anti-MRSA and anti-VRE Compounds and their Profiling for Cancer-Related Signaling Pathways

Mallika Kumarihamy[1], Siddharth Tripathi[1], Premalatha Balachandran[1], Bharathi Avula[1], Jianping Zhao[1], Mei Wang[2], Maria M. Bennett[1], Jin Zhang[1], Mary A. Carr[3]. K. Michael Lovell[3], Ocean I. Wellington[3], Mary E. Marquart[3], N. P. Dhammika Nanayakkara[1,*], Ilias Muhammad[1,*]

[1] National Center for Natural Products Research, Research Institute of Pharmaceutical Sciences, School of Pharmacy, University of Mississippi, University, Mississippi 38677

[2] Natural Products Utilization Research Unit, Agricultural Research Service, U.S. Department of Agriculture, University, Mississippi 38677

[3] Department of Cell and Molecular Biology, University of Mississippi Medical Center, 2500 North State Street, Jackson, MS 39216

***Correspondence**

Ilias Muhammad, National Center for Natural Products Research, Research Institute of Pharmaceutical Sciences, University of Mississippi, University, MS 38677, USA.

milias@olemiss.edu, Phone: +1-662-915-1051

N. P. Dhammika Nanayakkara, National Center for Natural Products Research, Research Institute of Pharmaceutical Sciences, University of Mississippi, University, MS 38677, USA.

dhammika@olemiss.edu; Phone: +1-662-915-1019

Figures S1-S48. NMR and HRMS spectra of compounds **1-17**

**Contents**	**Figure**
^{1}H NMR Spectrum of compound **1**	S1
^{13}C NMR Spectrum of compound **1**	S2
HRMS Spectrum of compound **1**	S3
^{1}H NMR Spectrum of compound **2**	S4
^{13}C NMR Spectrum of compound **2**	S5
HRMS Spectrum of compound **2**	S6
^{1}H NMR Spectrum of compound **3**	S7
^{13}C NMR Spectrum of compound **3**	S8
HRMS Spectrum of compound **3**	S9

^{1}H NMR Spectrum of compound **4**	S10
^{13}C NMR Spectrum of compound **4**	S11
HRMS Spectrum of compound **4**	S12
^{1}H NMR Spectrum of compound **5**	S13
^{13}C NMR Spectrum of compound **5**	S14
HRMS Spectrum of compound **5**	S15
^{1}H NMR Spectrum of compound **6**	S16
^{13}C NMR Spectrum of compound **6**	S17
HRMS Spectrum of compound **6**	S18
^{1}H NMR Spectrum of compound **7**	S19
^{13}C NMR Spectrum of compound **7**	S20
HRMS Spectrum of compound **7**	S21
^{1}H NMR Spectrum of compound **8**	S22
^{13}C NMR Spectrum of compound **8**	S23
HRMS Spectrum of compound **8**	S24
^{1}H NMR Spectrum of compounds **9** & **10**	S25
^{13}C NMR Spectrum of compounds **9** & **10**	S26
HRMS Spectrum of compounds **9** & **10**	S27
^{1}H NMR Spectrum of compound **11**	S28
^{13}C NMR Spectrum of compound **11**	S29
HRMS Spectrum of compound **11**	S30
^{1}H NMR Spectrum of compound **12**	S31
^{13}C NMR Spectrum of compound **12**	S32
HRMS Spectrum of compound **12**	S33
^{1}H NMR Spectrum of compound **13**	S34
^{13}C NMR Spectrum of compound **13**	S35
HRMS Spectrum of compound **13**	S36
^{1}H NMR Spectrum of compound **14**	S37
^{13}C NMR Spectrum of compound **14**	S38
HRMS Spectrum of compound **14**	S39
^{1}H NMR Spectrum of compound **15**	S40
^{13}C NMR Spectrum of compound **15**	S41
HRMS Spectrum of compound **15**	S42
^{1}H NMR Spectrum of compound **16**	S43
^{13}C NMR Spectrum of compound **16**	S44
HRMS Spectrum of compound **16**	S45
^{1}H NMR Spectrum of compound **17**	S46
^{13}C NMR Spectrum of compound **17**	S47
HRMS Spectrum of compound **17**	S48
List of luciferase vectors	Table S1

Compound **1**

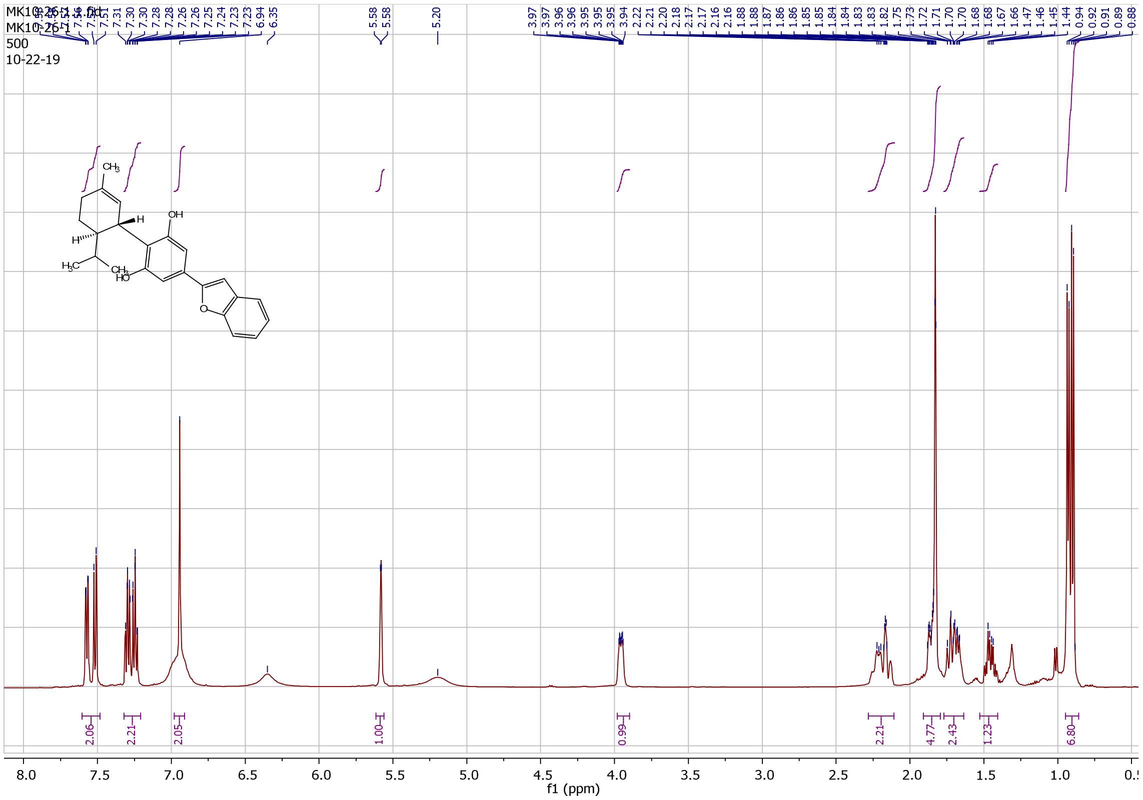

Figure S1. ^{1}H NMR (CD_3OD, 500 MHz) of **1.**

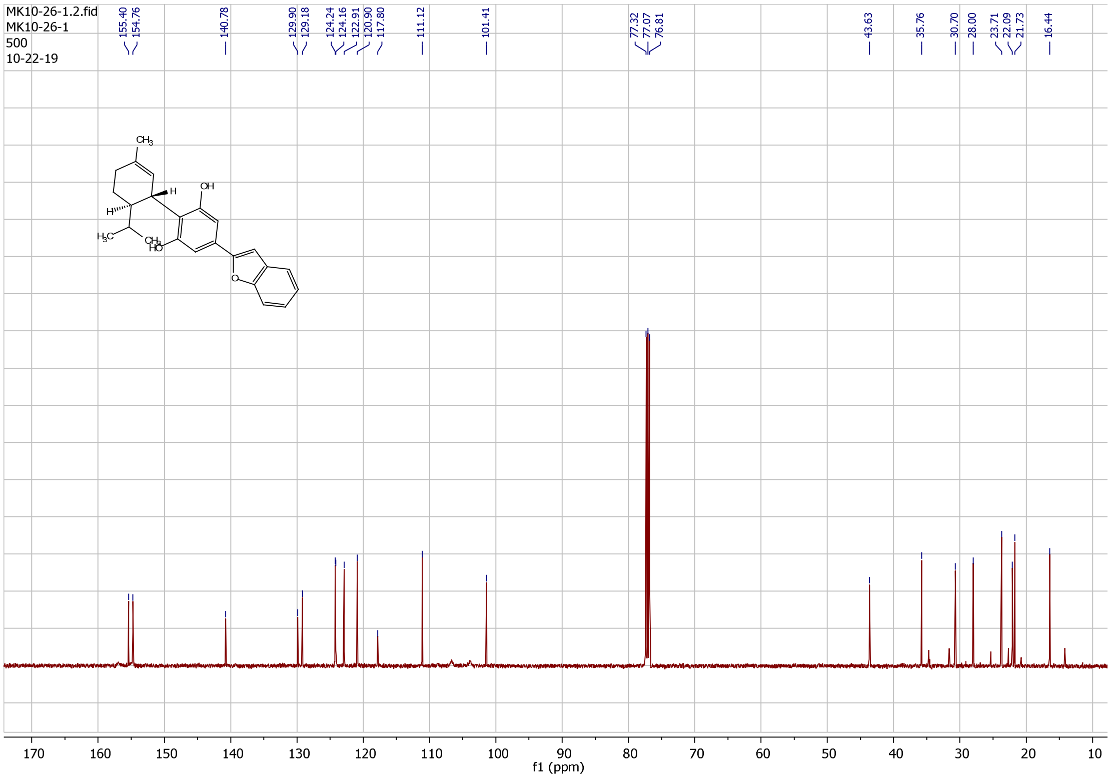

Figure S2. ^{13}C NMR (CD_3OD, 126 MHz) of **1.**

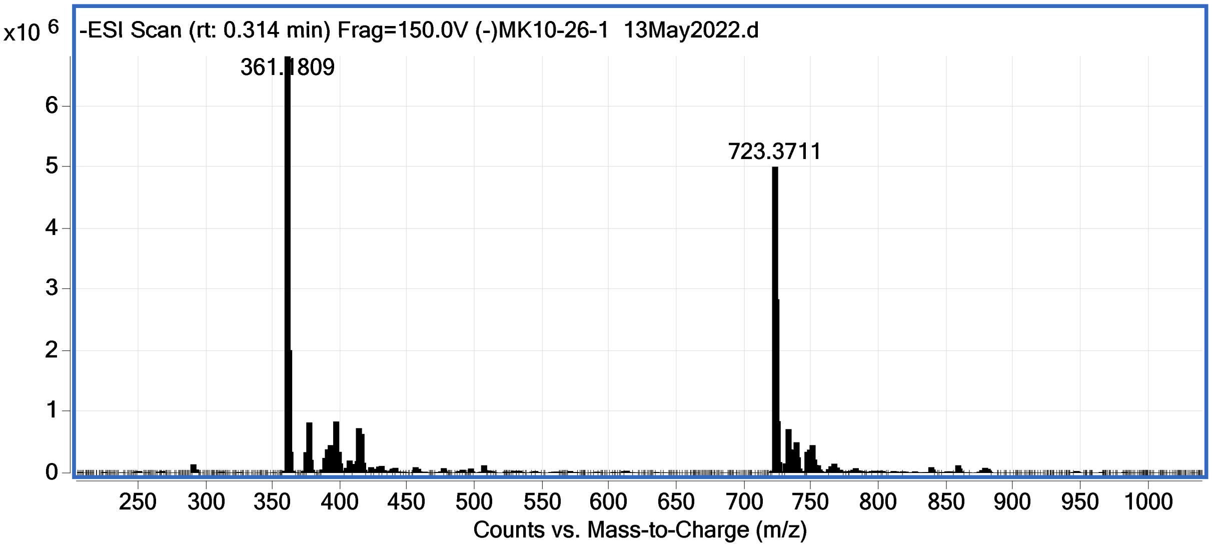

Figure S3. HRMS data of **1.**

Compound **2**

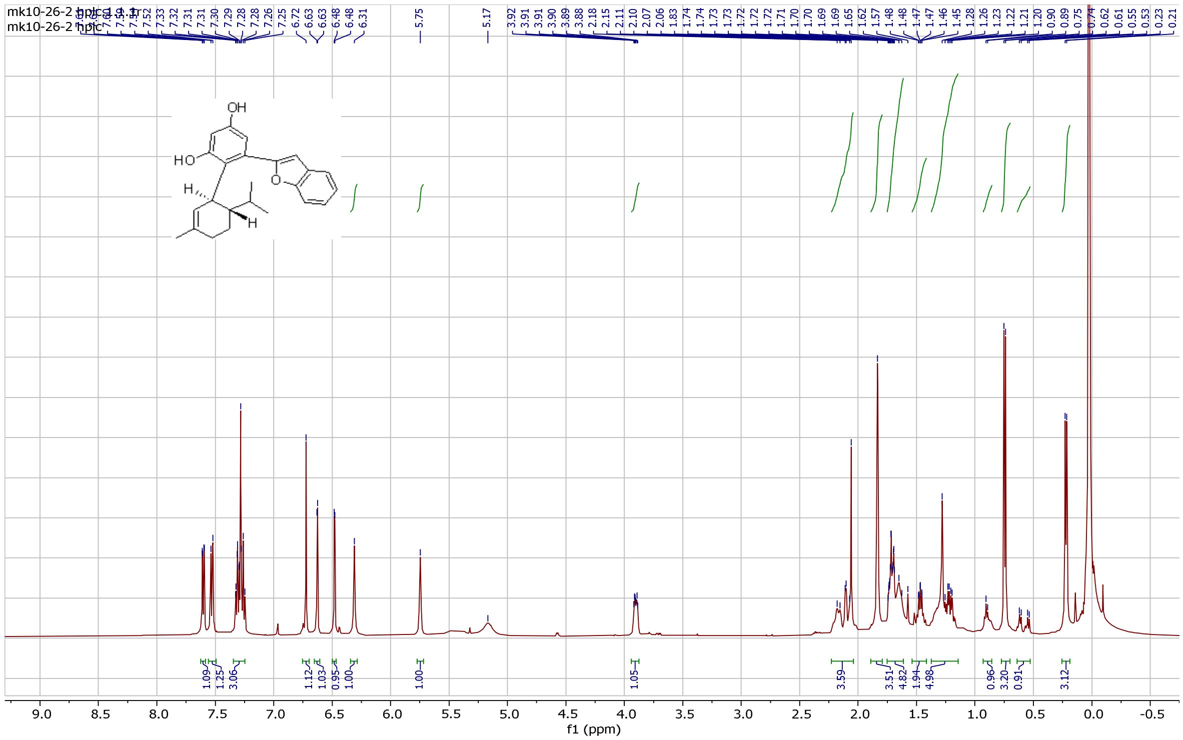

Figure S4. ^{1}H NMR (CD_3OD, 400 MHz) of **2.**

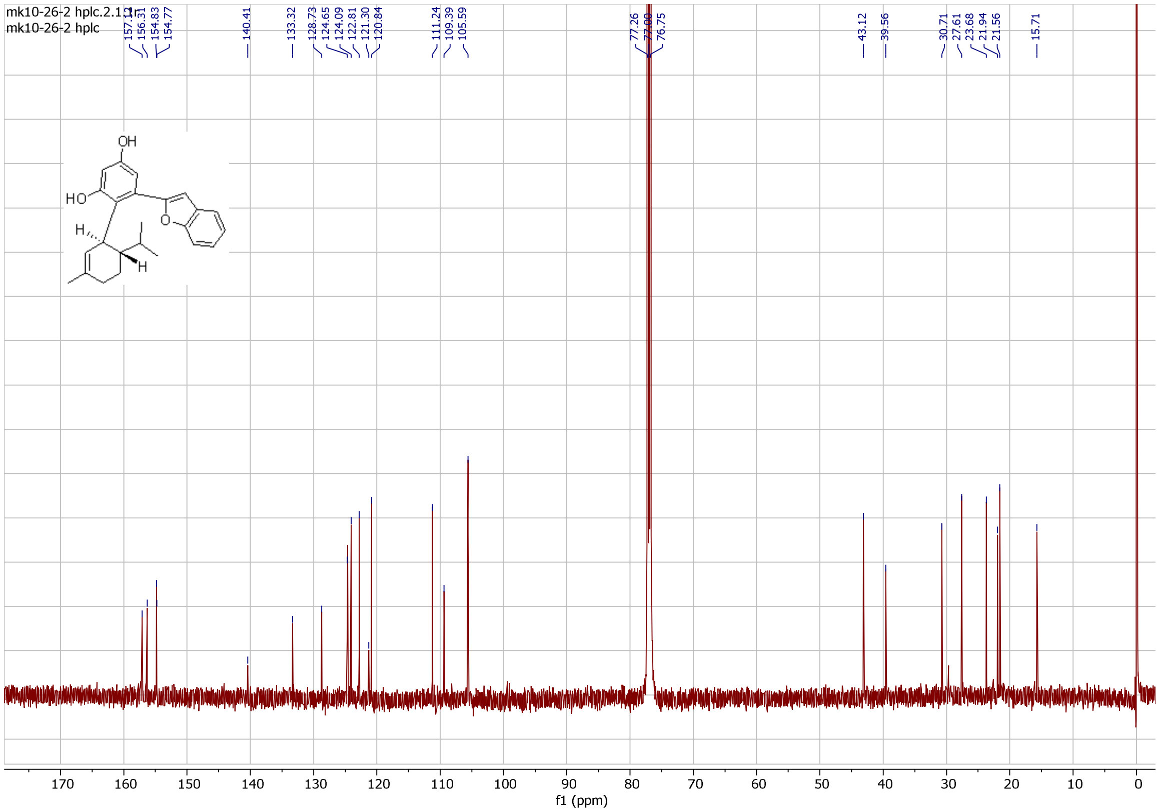

Figure S5. ^{13}C NMR (CD_3OD, 100 MHz) of **2.**

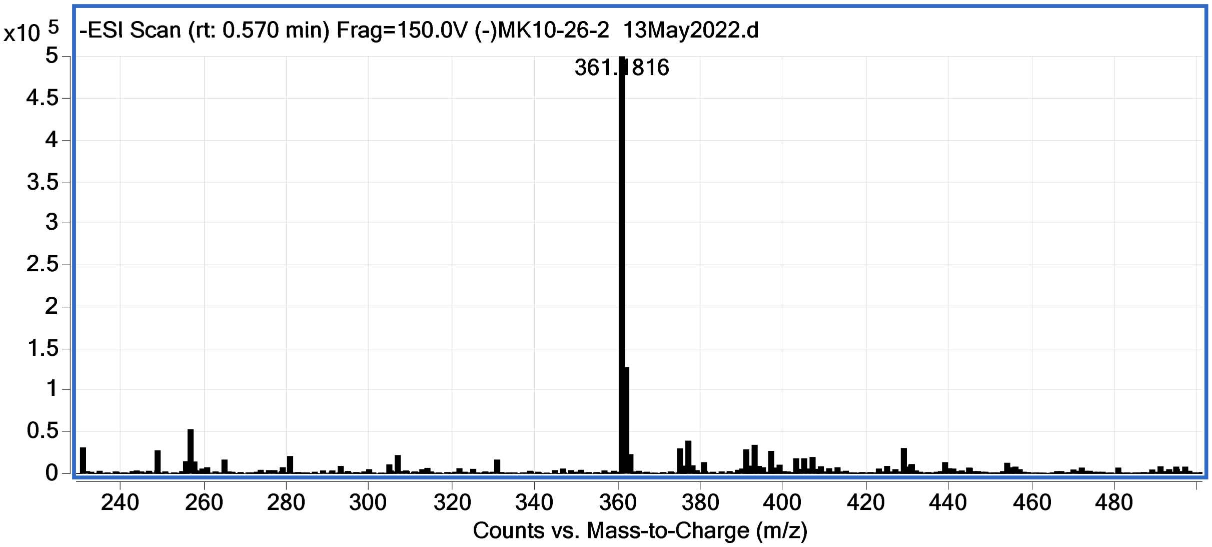

Figure S6. HRMS data of **2.**

Compound **3**

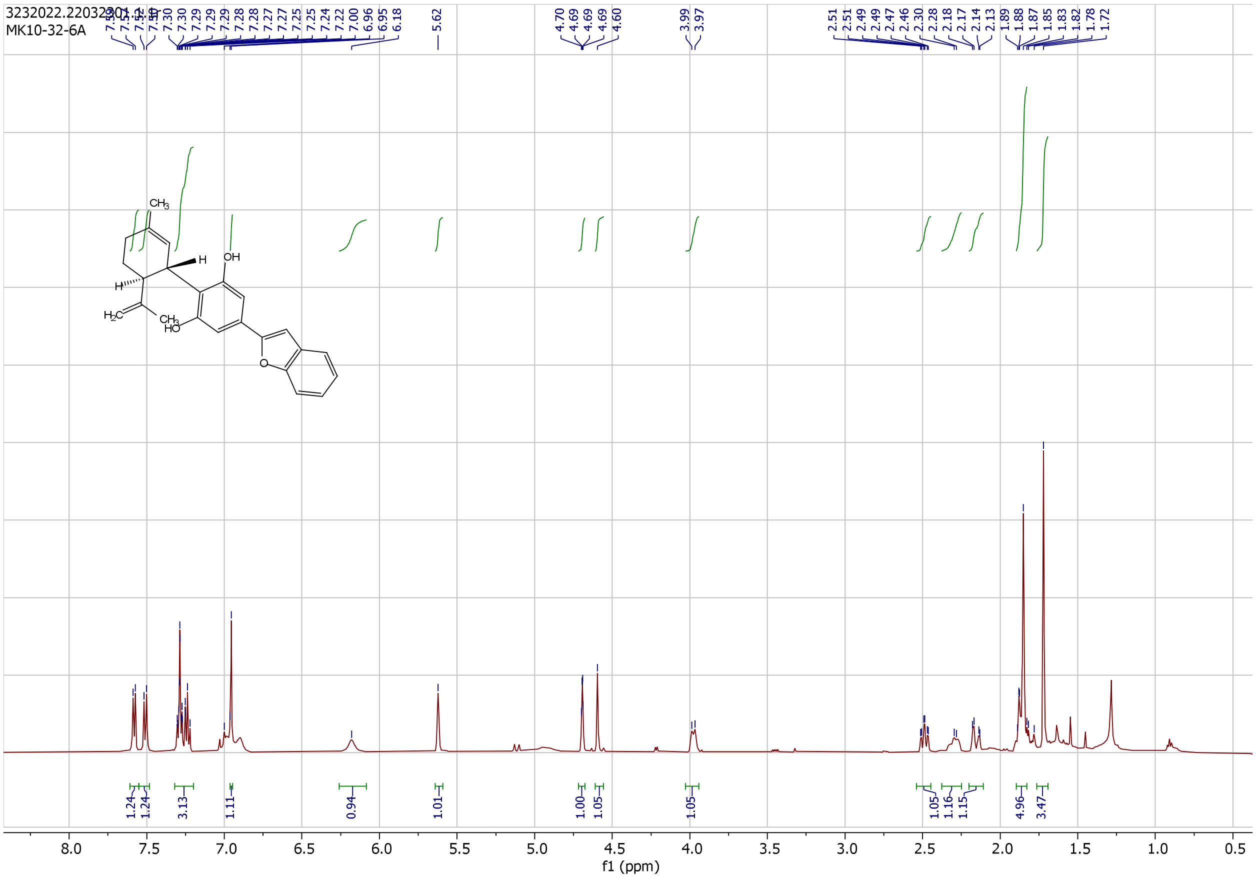

Figure S7. ^1H NMR (CD_3OD, 500 MHz) of **3.**

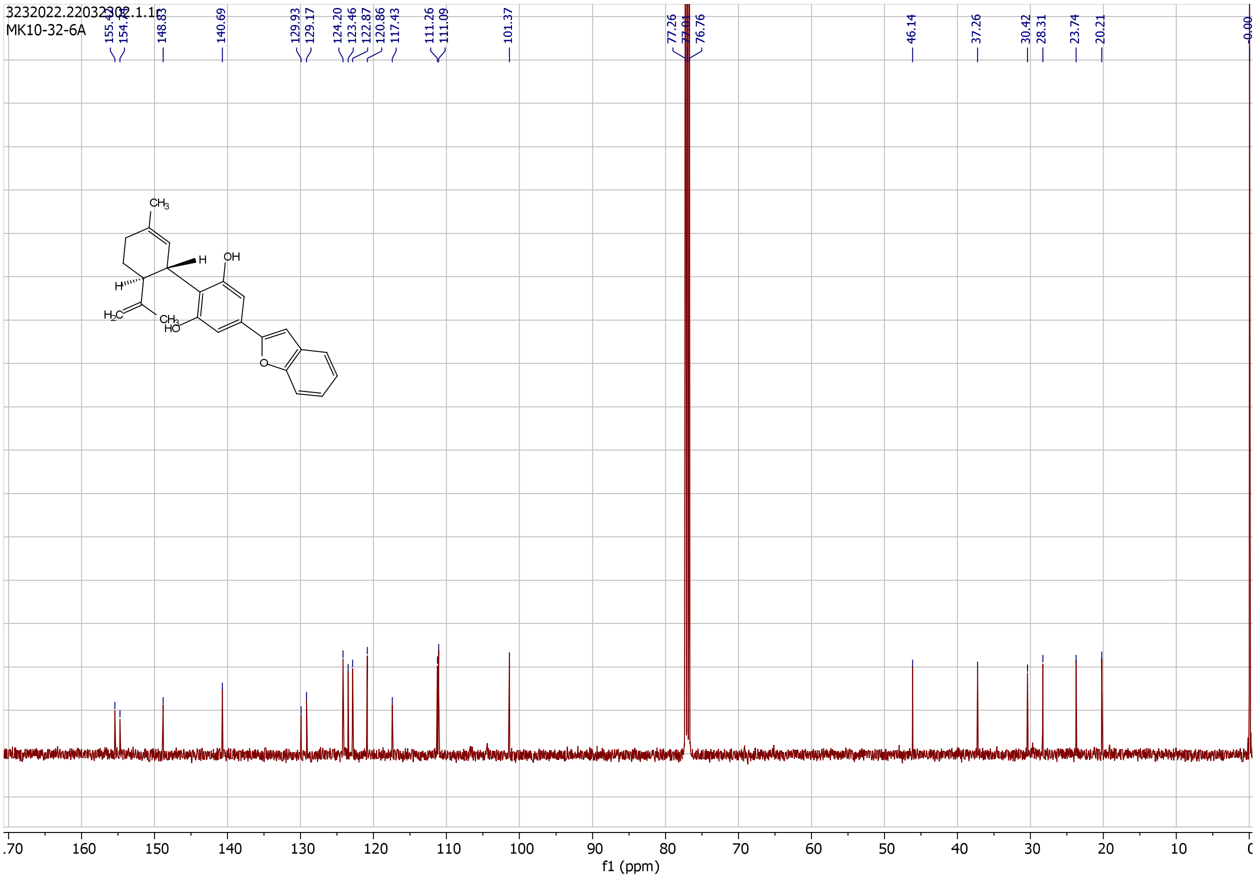

Figure S8. ^{13}C NMR (CD_3OD, 126 MHz) of **3.**

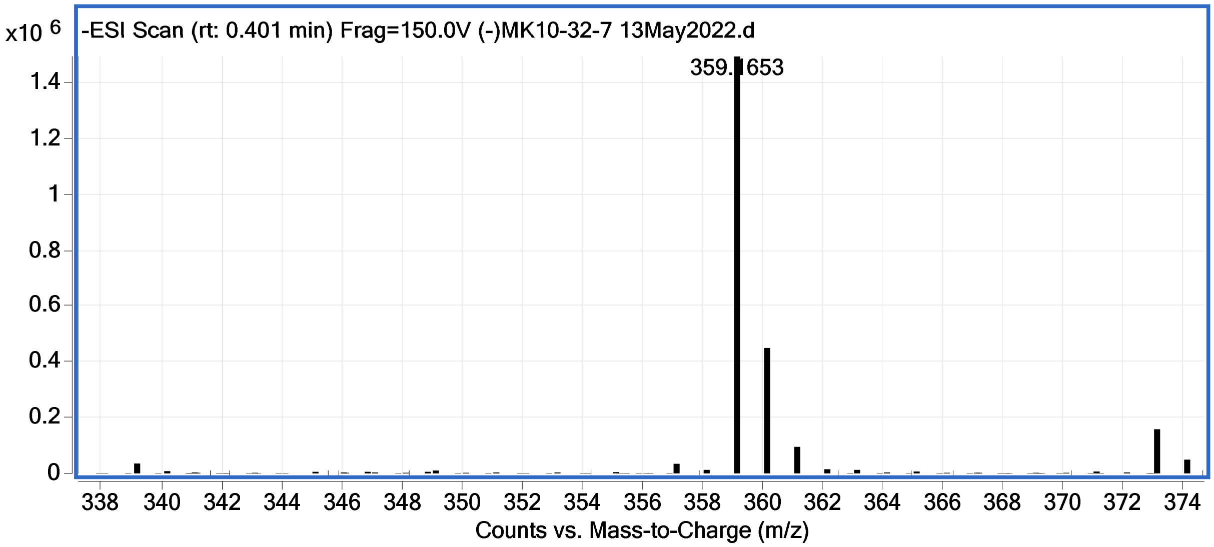

Figure S9. HRMS data of 3.

Compound **4**

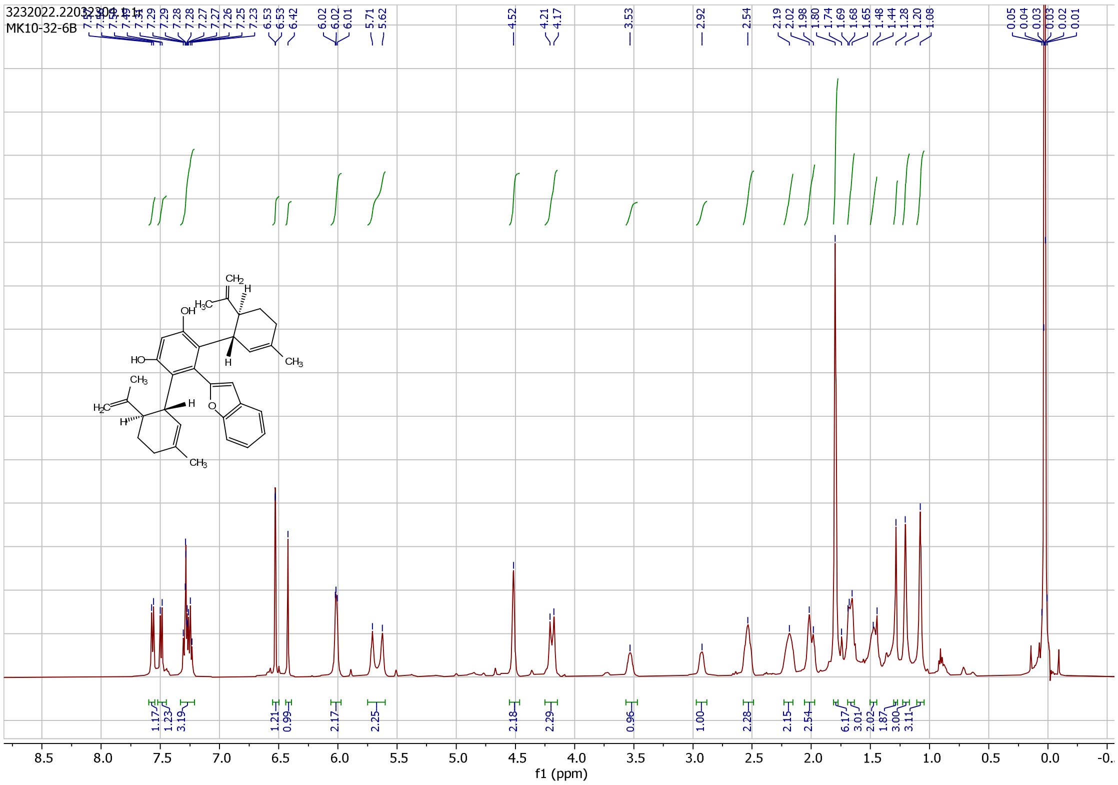

Figure S10. ^{1}H NMR (CD_3OD, 500 MHz) of **4.**

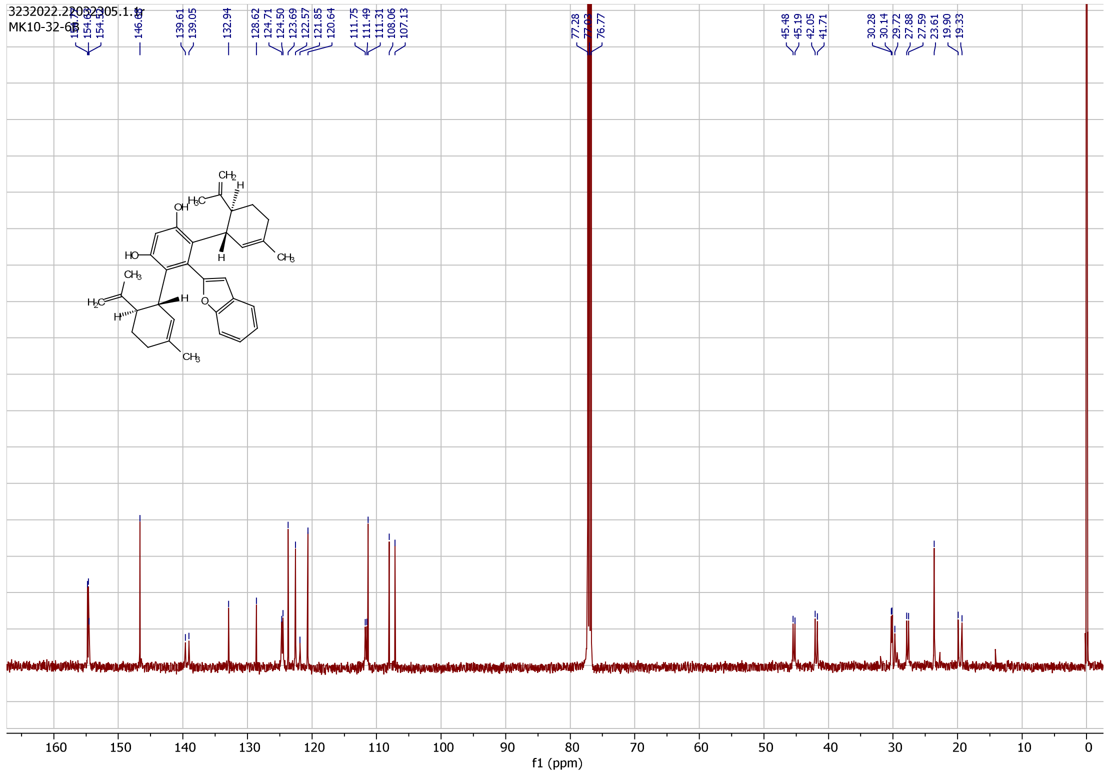

Figure S11. ^{13}C NMR (CD_3OD, 126 MHz) of **4.**

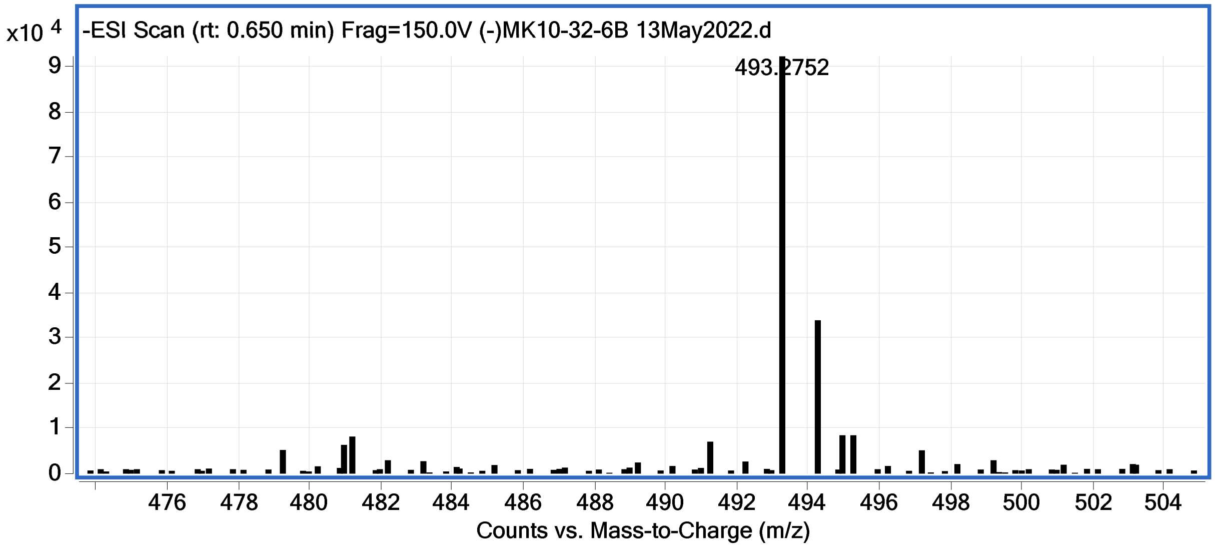

Figure S12. HRMS data of 4.

Compound **5**

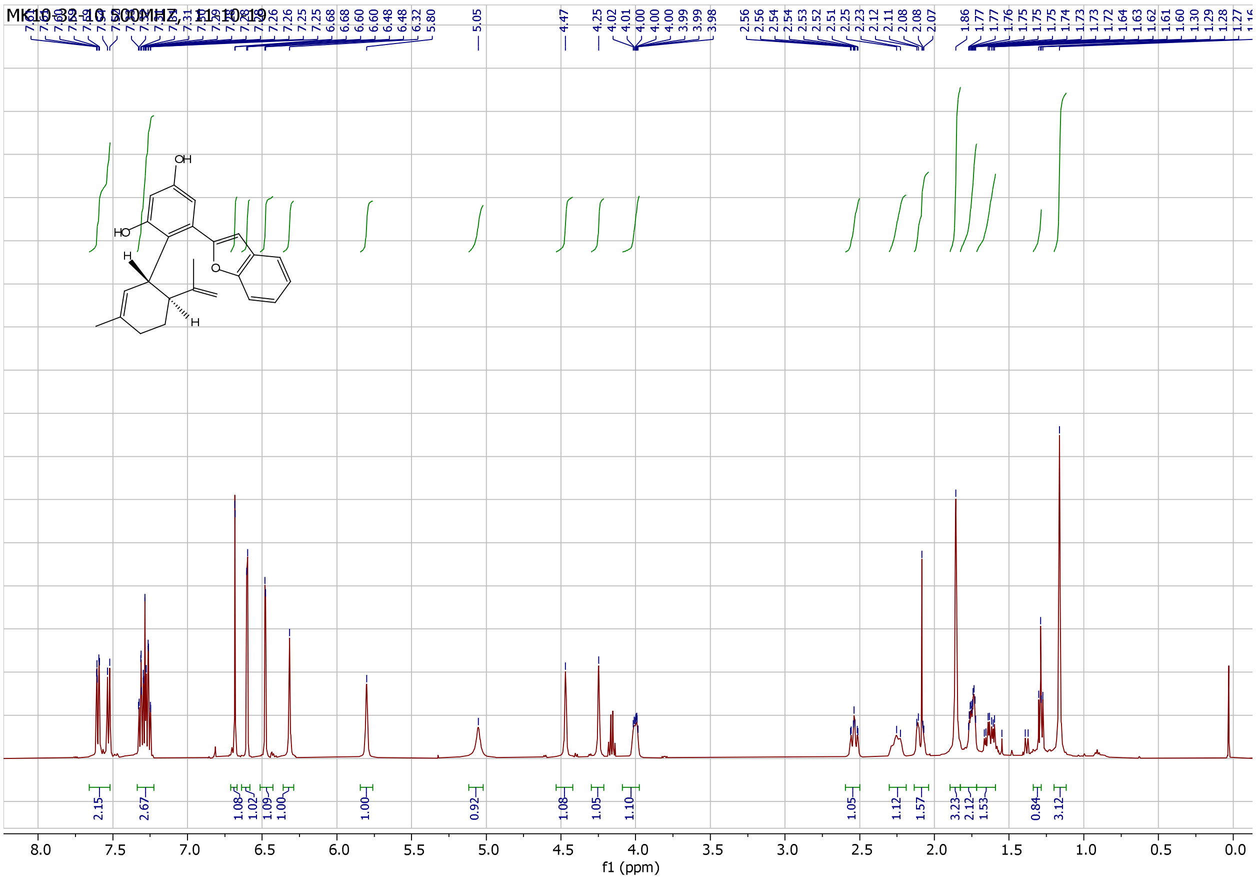

Figure S13. ^{1}H NMR (500 MHz, Chloroform-*d*) of **5.**

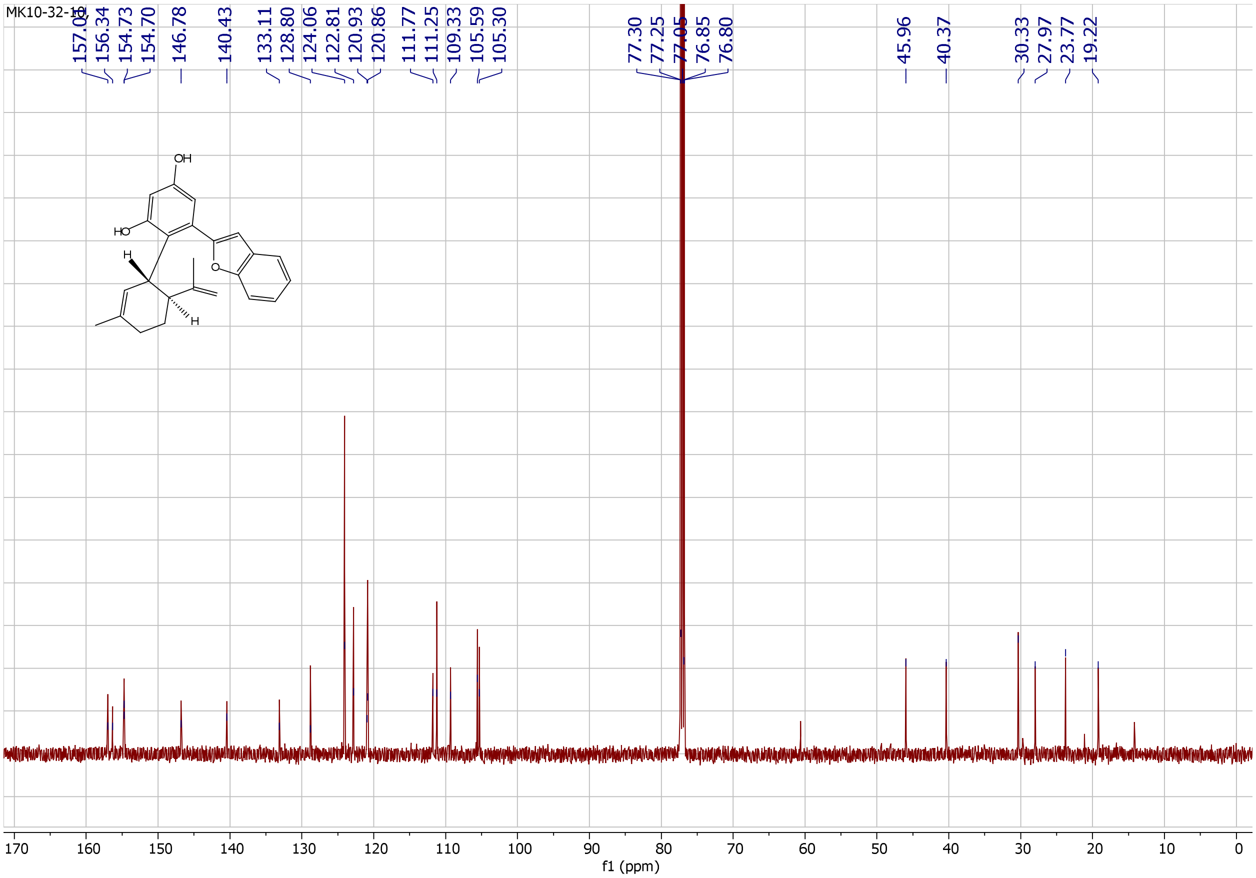

Figure S14. ^{13}C NMR (126 MHz, Chloroform-*d*) of **5.**

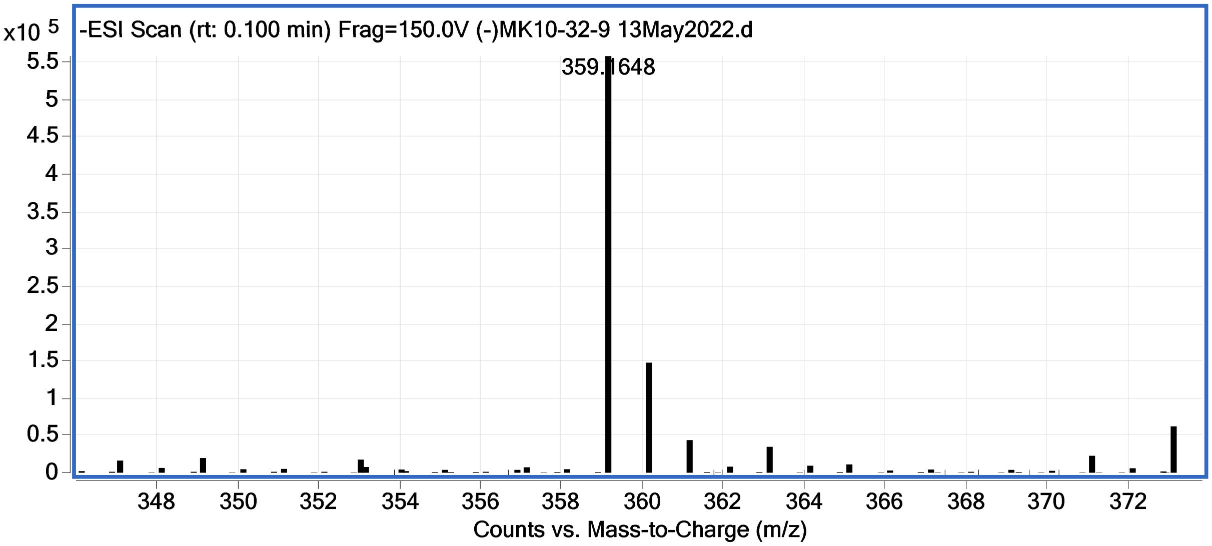

Figure S15. HRMS data of 5.

Compound **6**

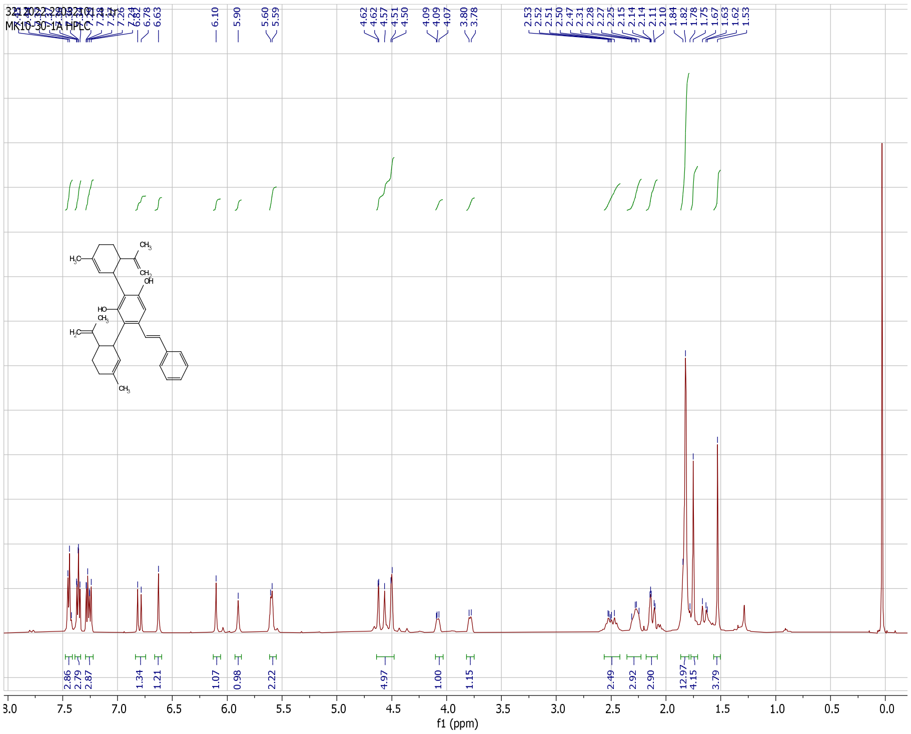

Figure S16. ^{1}H NMR (500 MHz, Chloroform-*d*) of **6.**

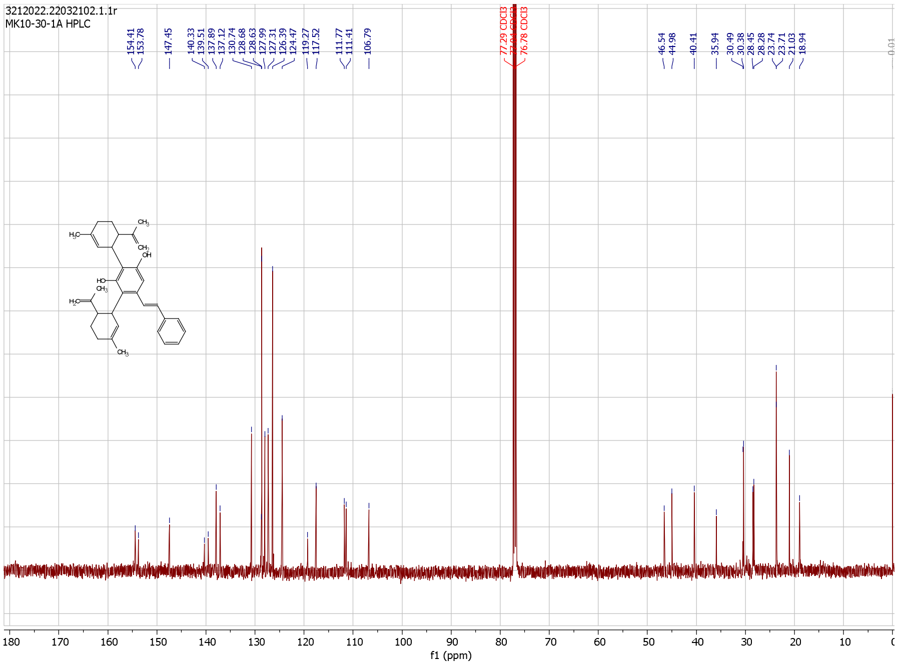

Figure S17. ^{13}C NMR (126 MHz, Chloroform-*d*) of **6.**

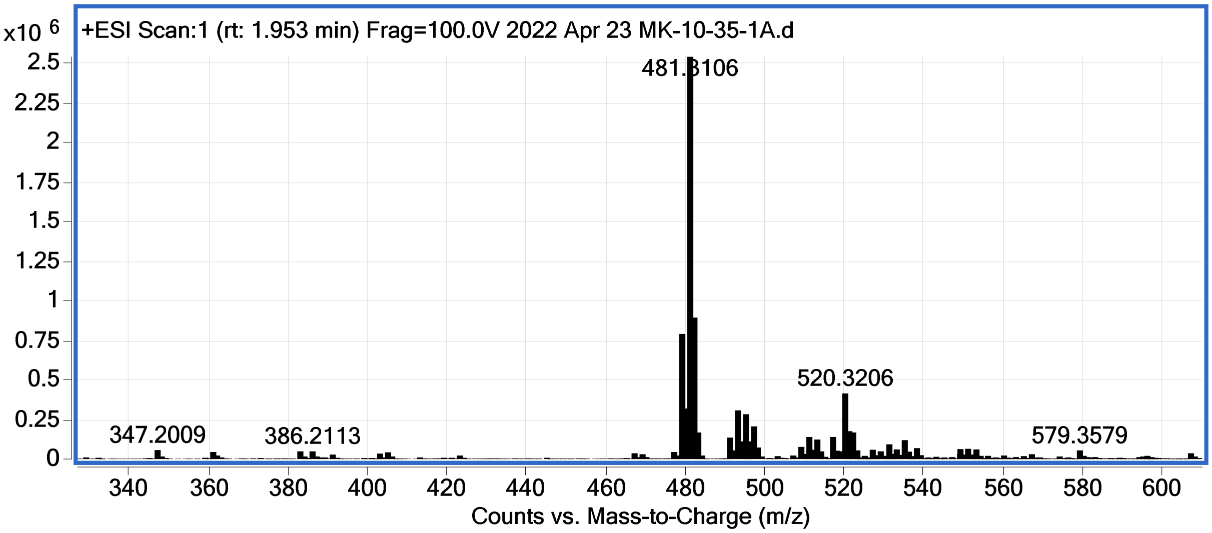

Figure S18. HRMS data of 6.

Compound **7**

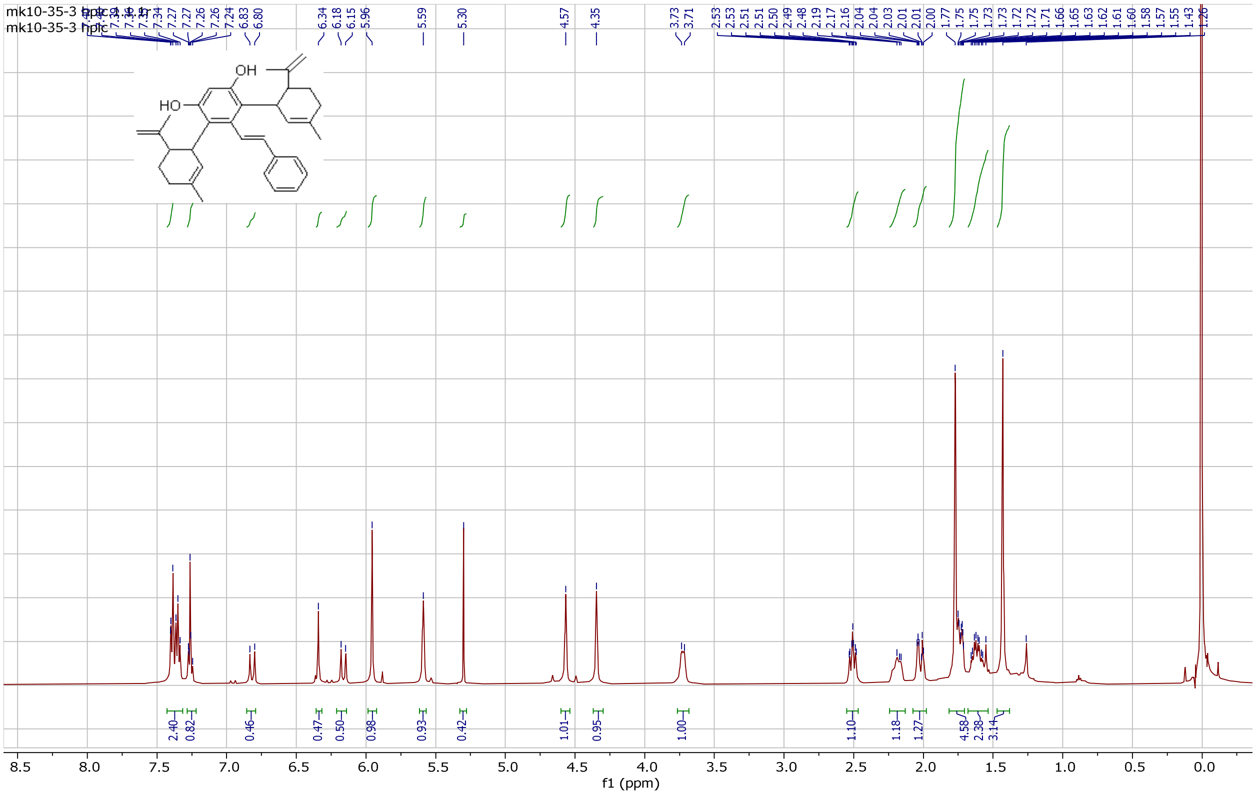

Figure S19. ^{1}H NMR (500 MHz, Chloroform-*d*) of **7.**

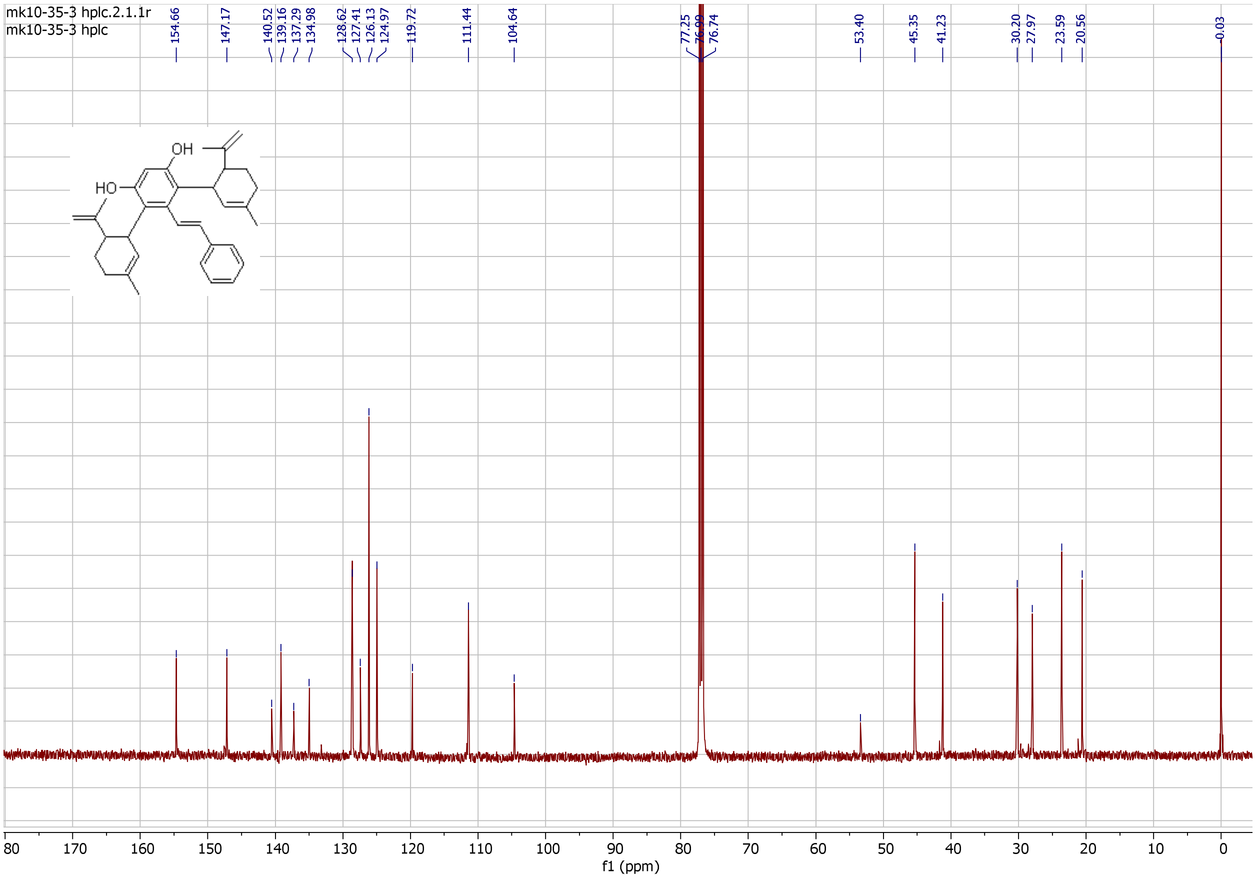

Figure S20. ^{13}C NMR (126 MHz, Chloroform-*d*) of **7.**

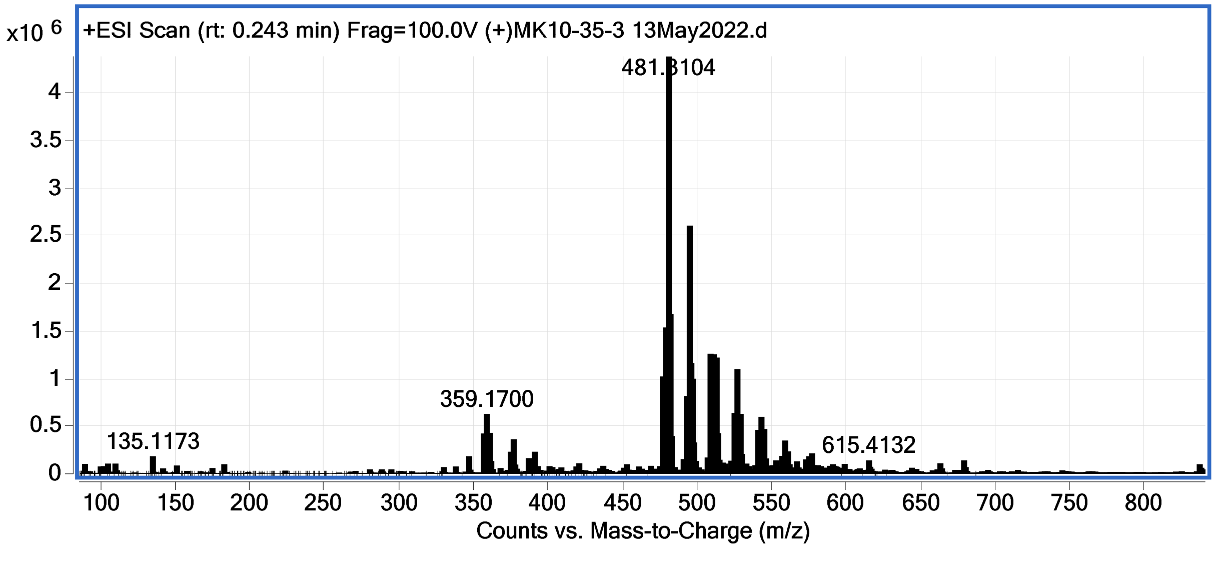

Figure S21. HRMS data of 7.

Compound **8**

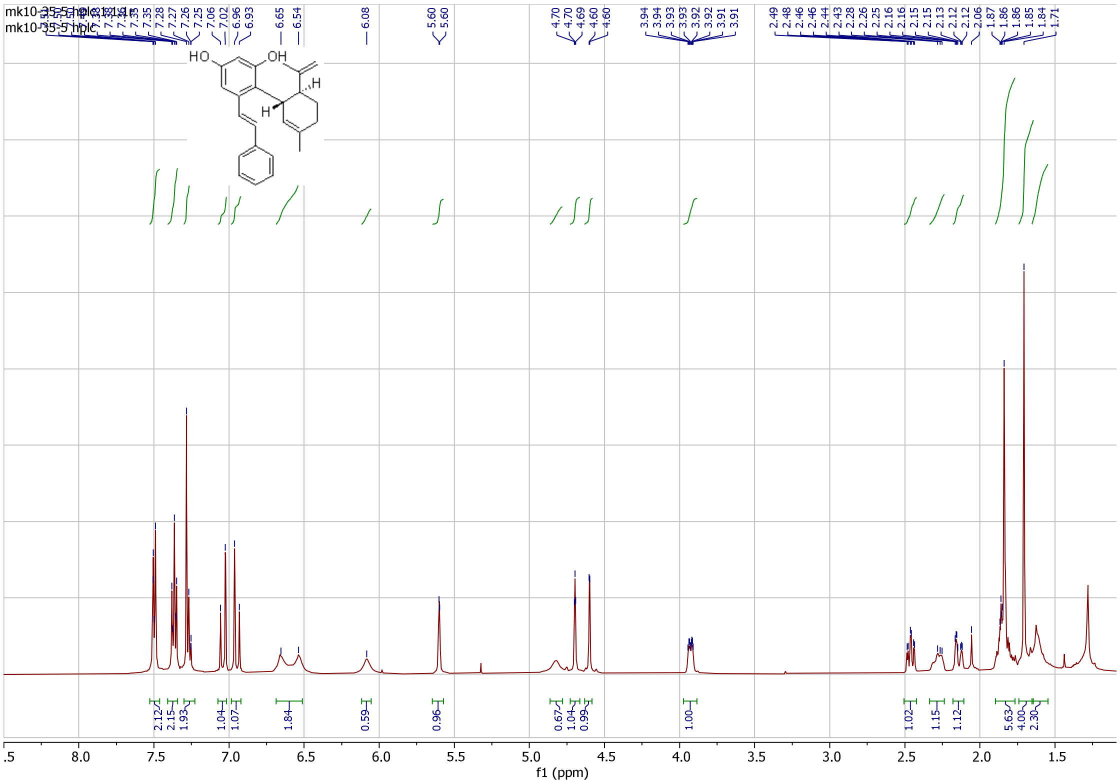

Figure S22. ^{1}H NMR (500 MHz, Chloroform-*d*) of **8.**

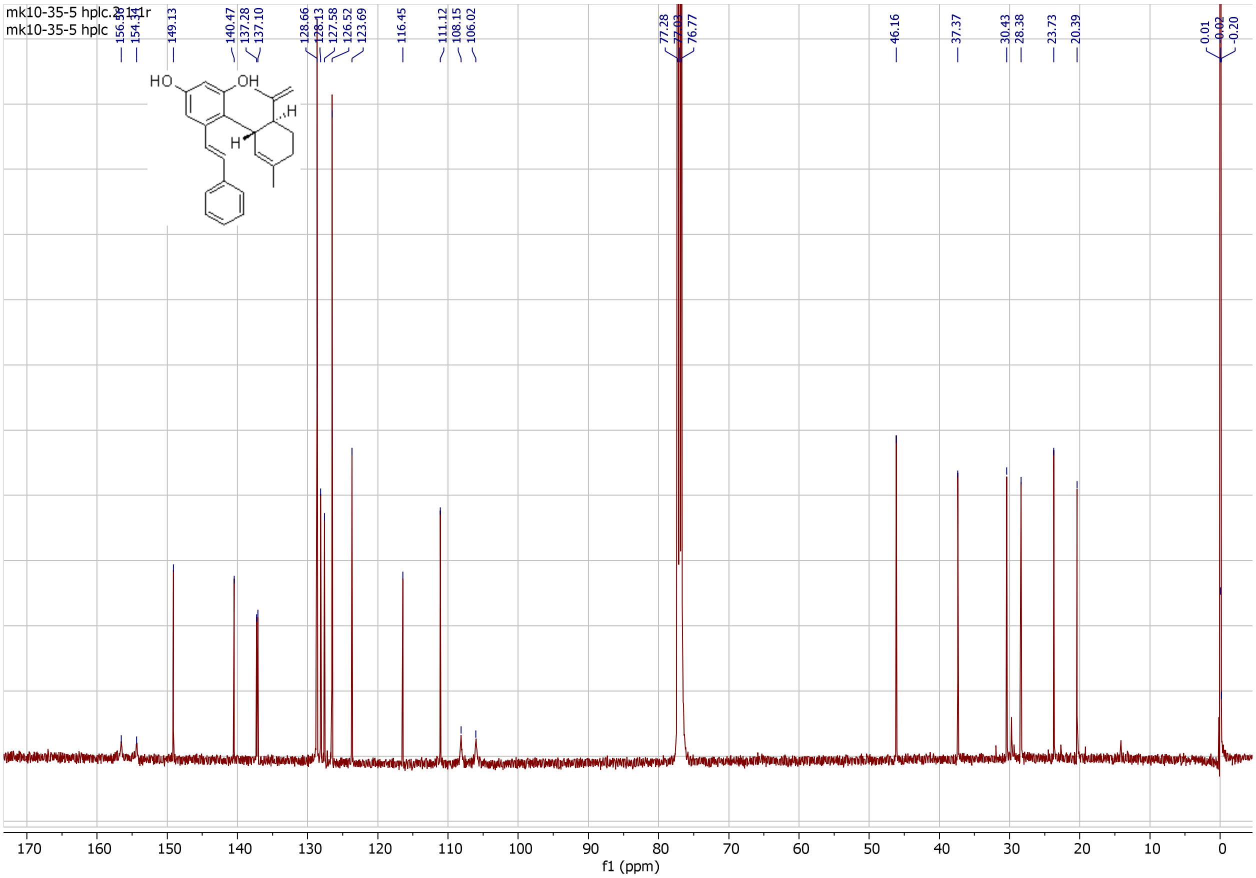

Figure S23. ^{13}C NMR (126 MHz, Chloroform-*d*) of **8.**

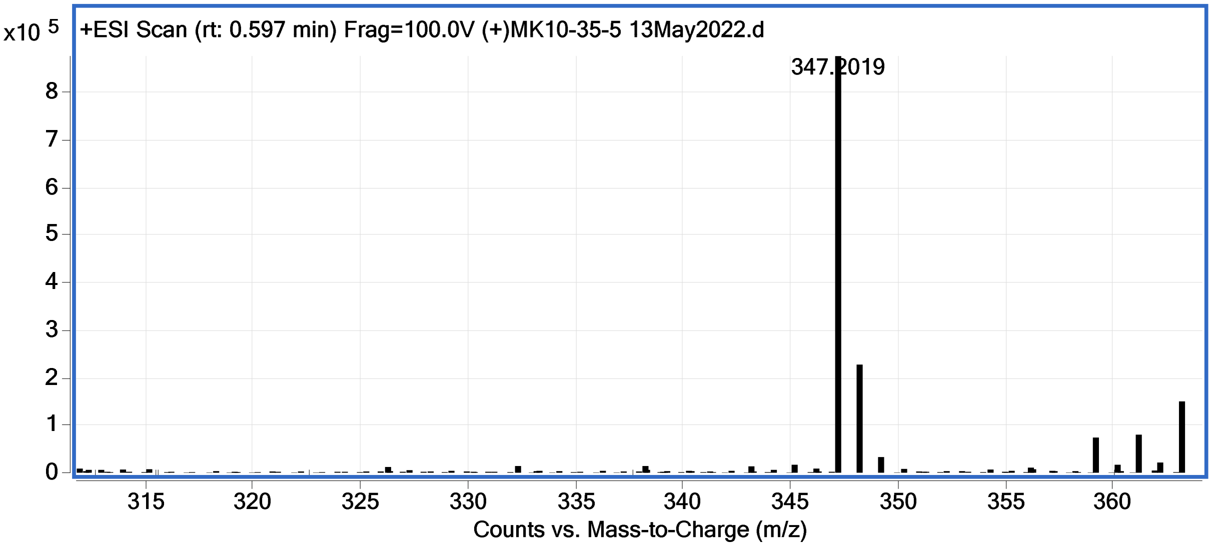

Figure S24. HRMS data of 8.

Compound **9** & **10** **(1: 0.2** mixture**)**

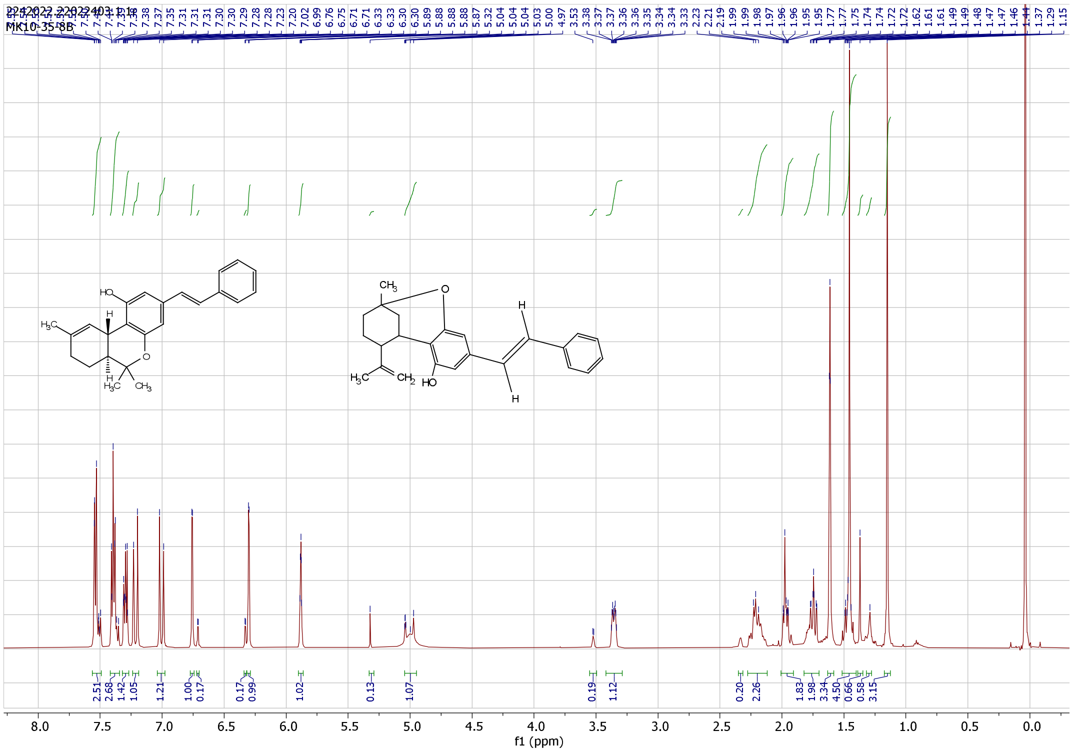

Figure S25. ^{1}H NMR (500 MHz, Chloroform-*d*) of **9** & **10.**

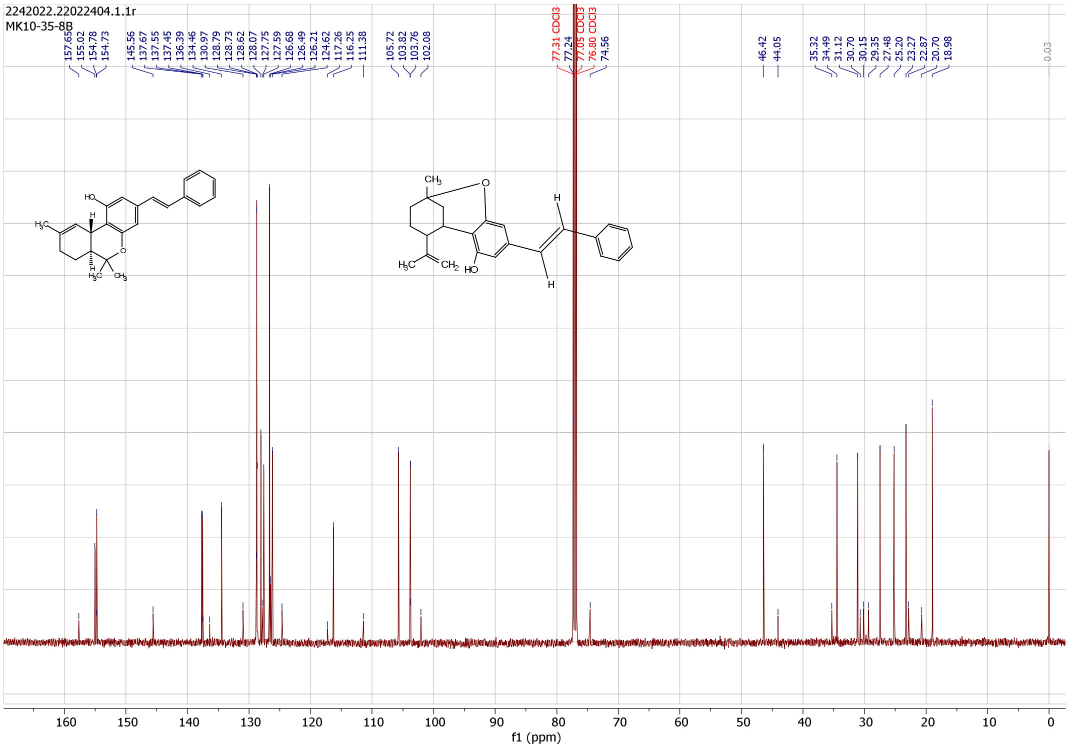

Figure S26. ^{13}C NMR (126 MHz, Chloroform-*d*) of **9** & **10.**

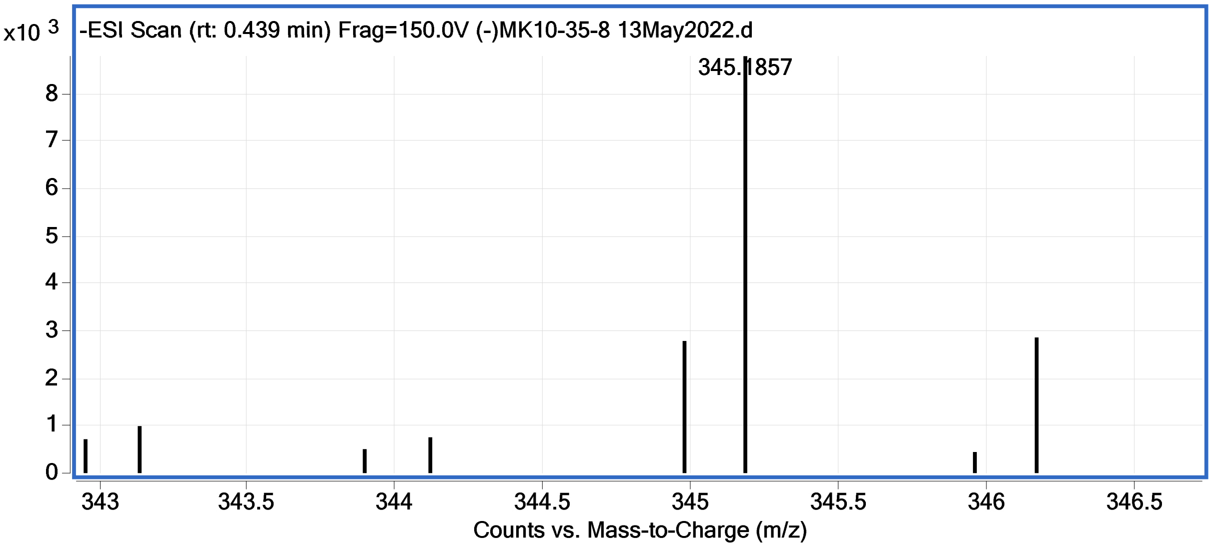

Figure S27. HRMS data of 9 & 10.

Compound **11**

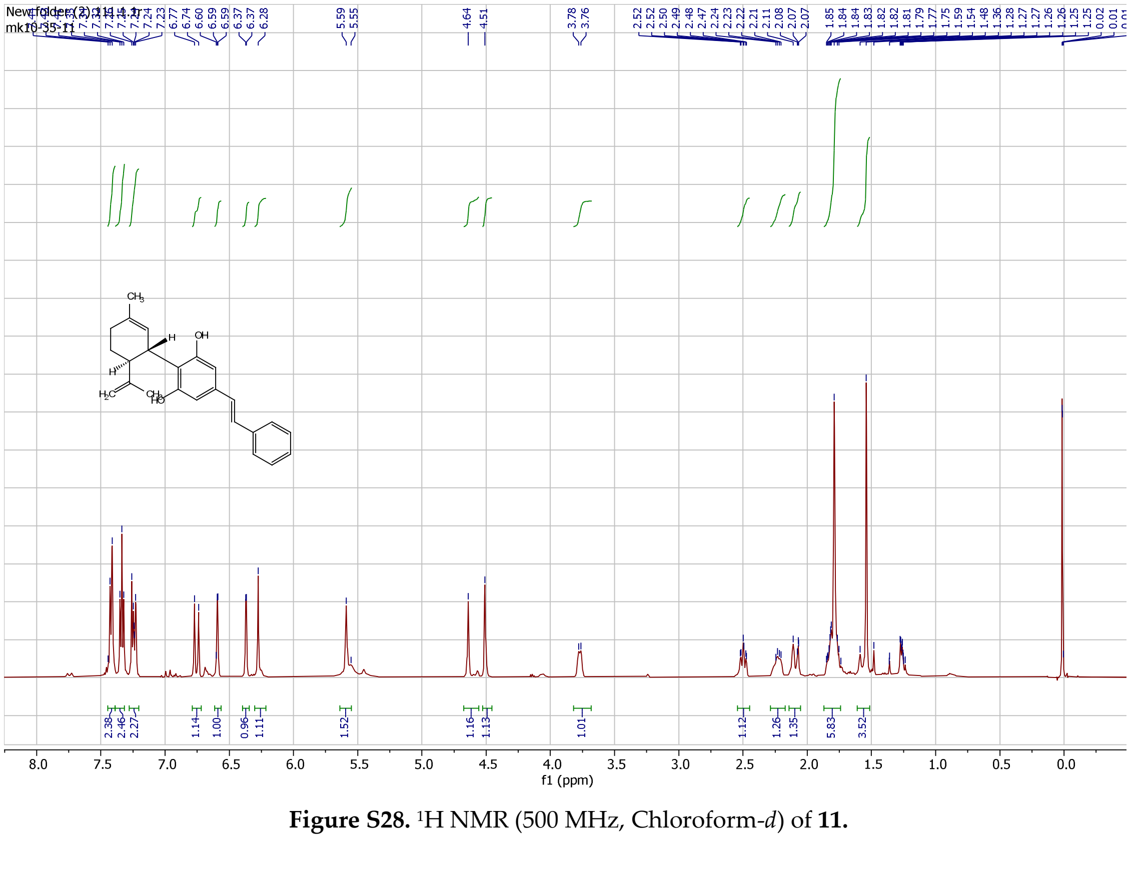

Figure S28. ^{1}H NMR (500 MHz, Chloroform-*d*) of **11.**

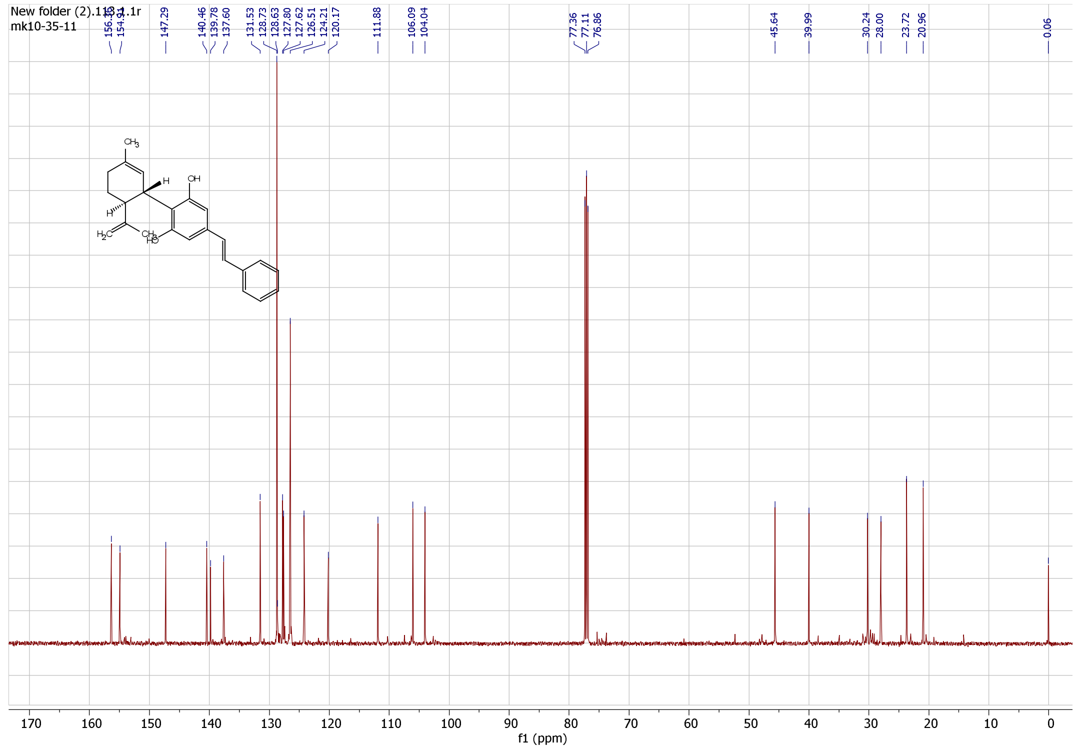

Figure S29. ^{13}C NMR (126 MHz, Chloroform-*d*) of **11.**

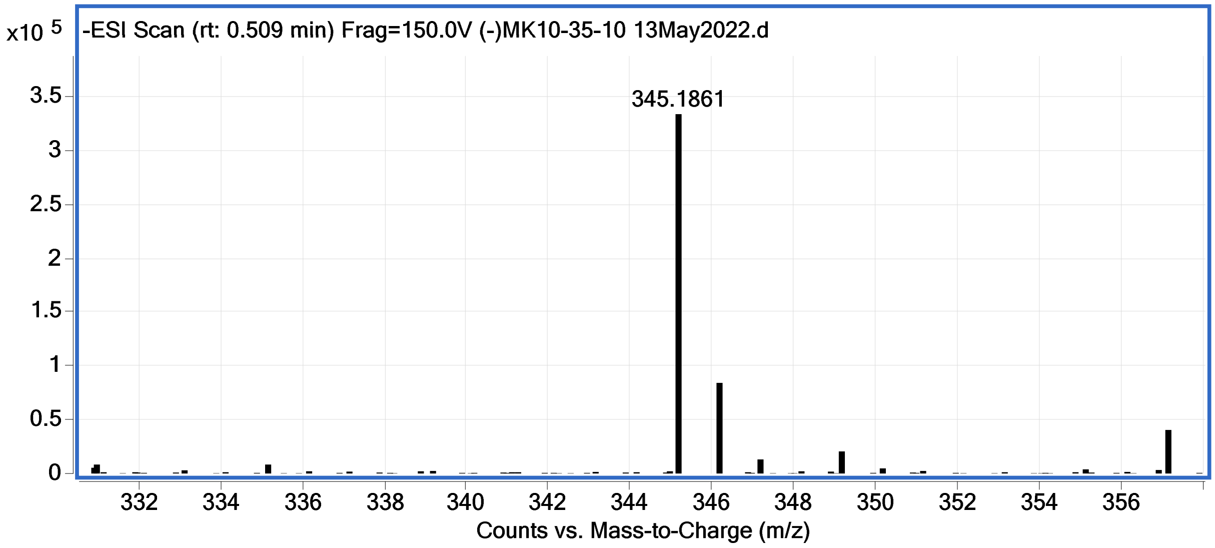

Figure S30. HRMS data of 11.

Compound **12**

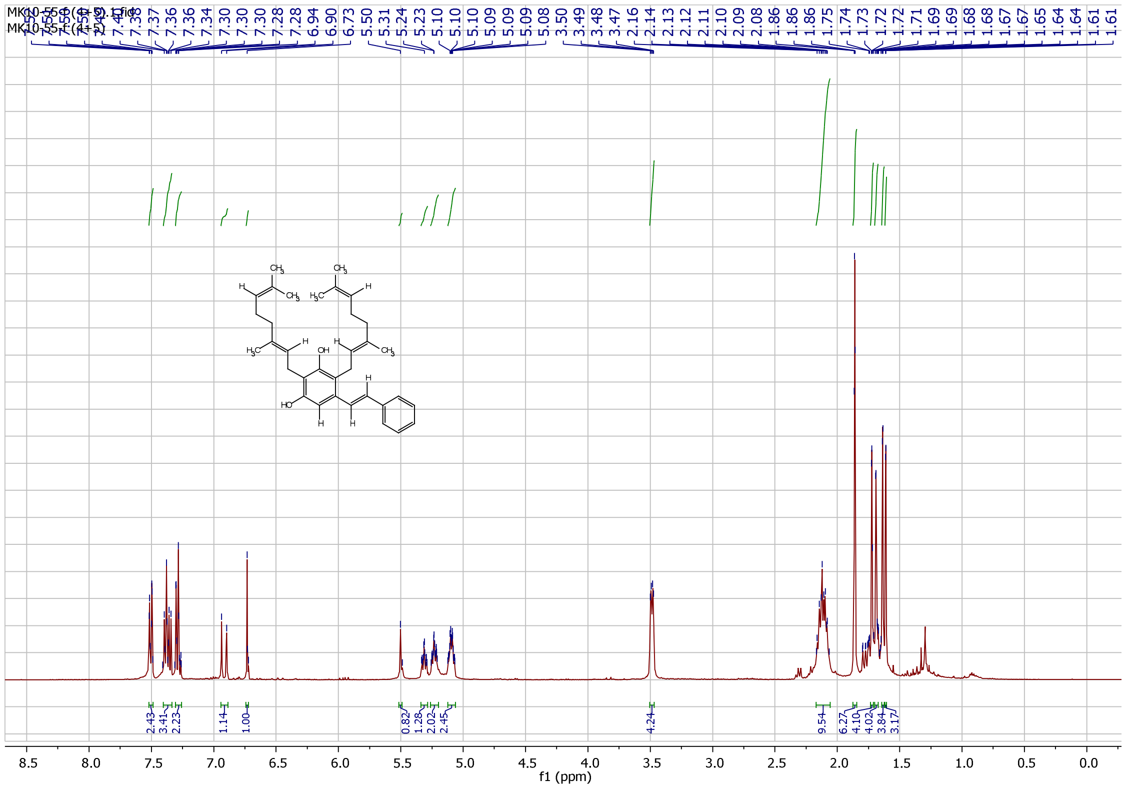

Figure S31. ^{1}H NMR (400 MHz, Chloroform-*d*) of **12.**

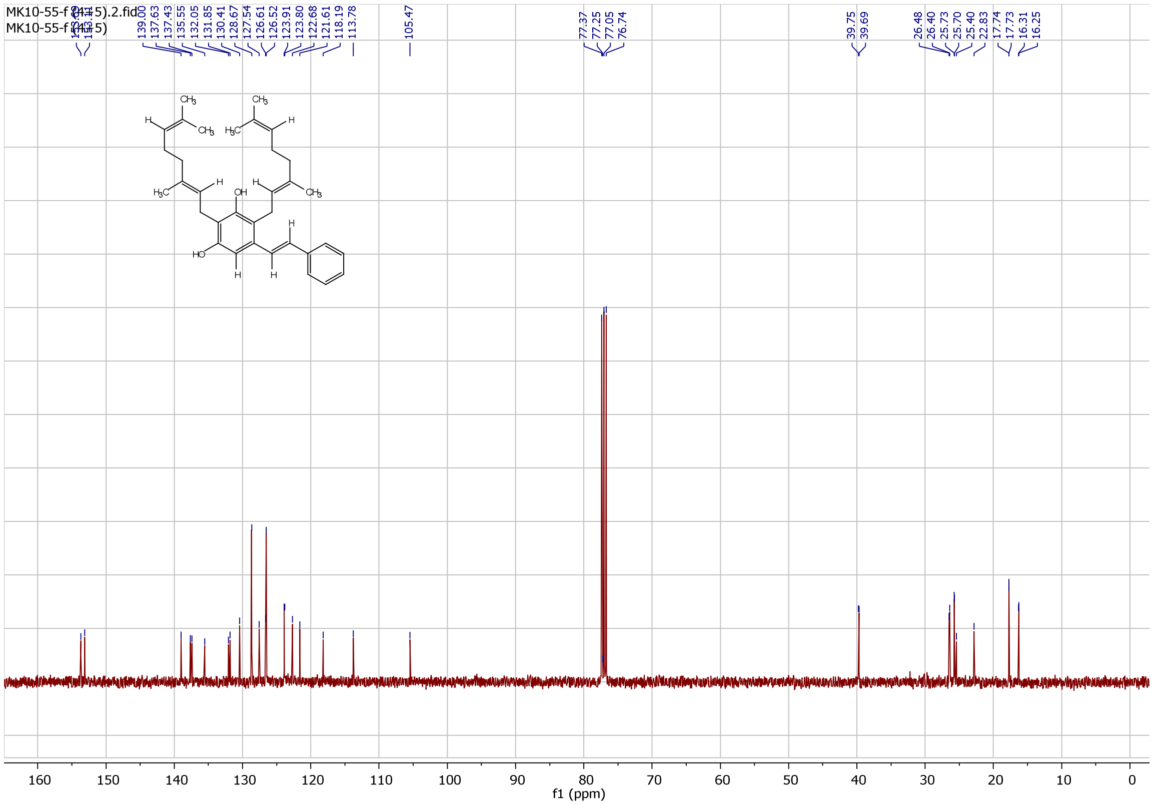

Figure S32. ^{13}C NMR (101 MHz, Chloroform-*d*) of **12.**

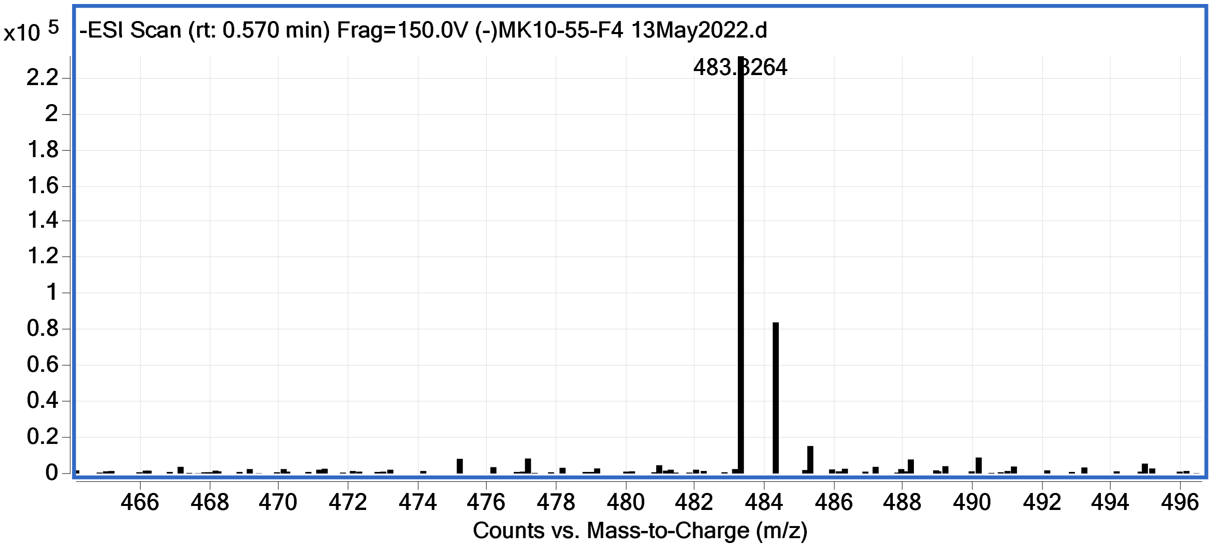

Figure S33. HRMS data of 12.

Compound **13**

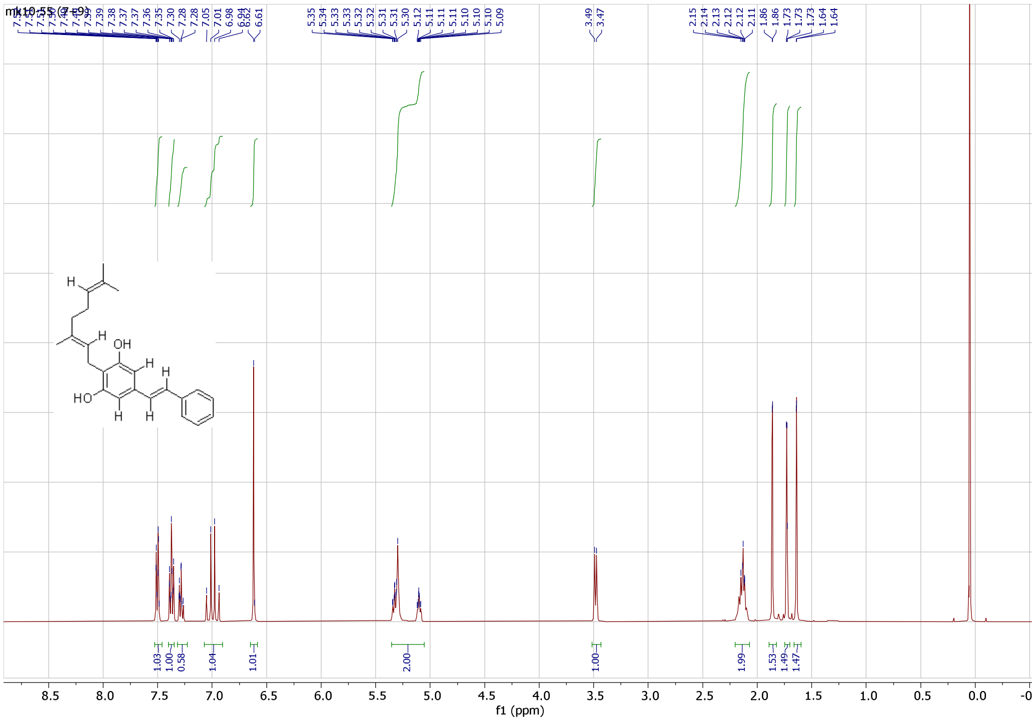

Figure S34. ^{1}H NMR (400 MHz, Chloroform-*d*) of **13.**

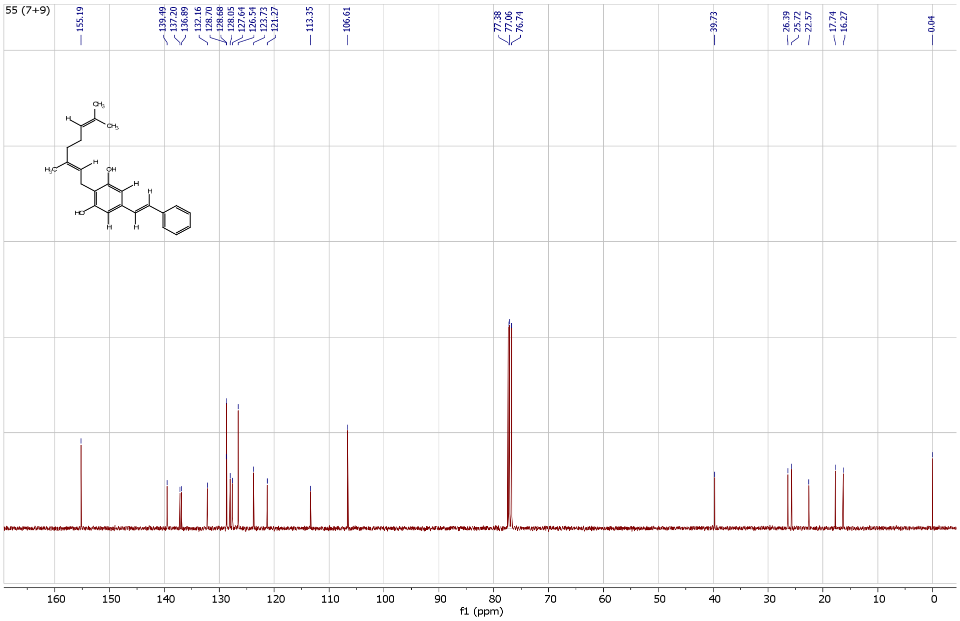

Figure S35. ^{13}C NMR (101 MHz, $CDCl_3$) of **13.**

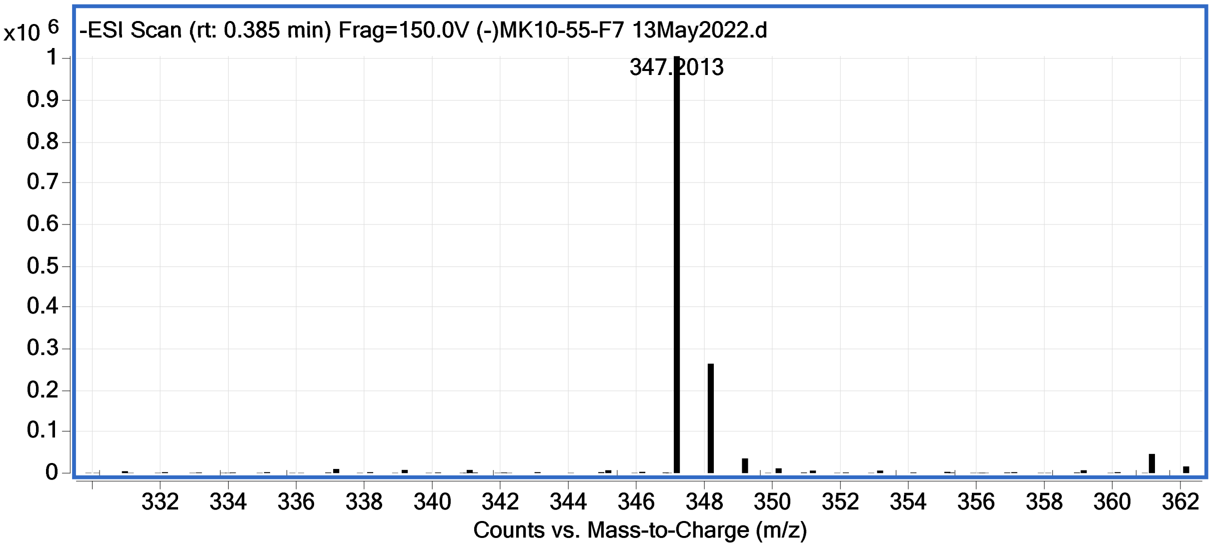

Figure S36. HRMS data of 13.

Compound **14**

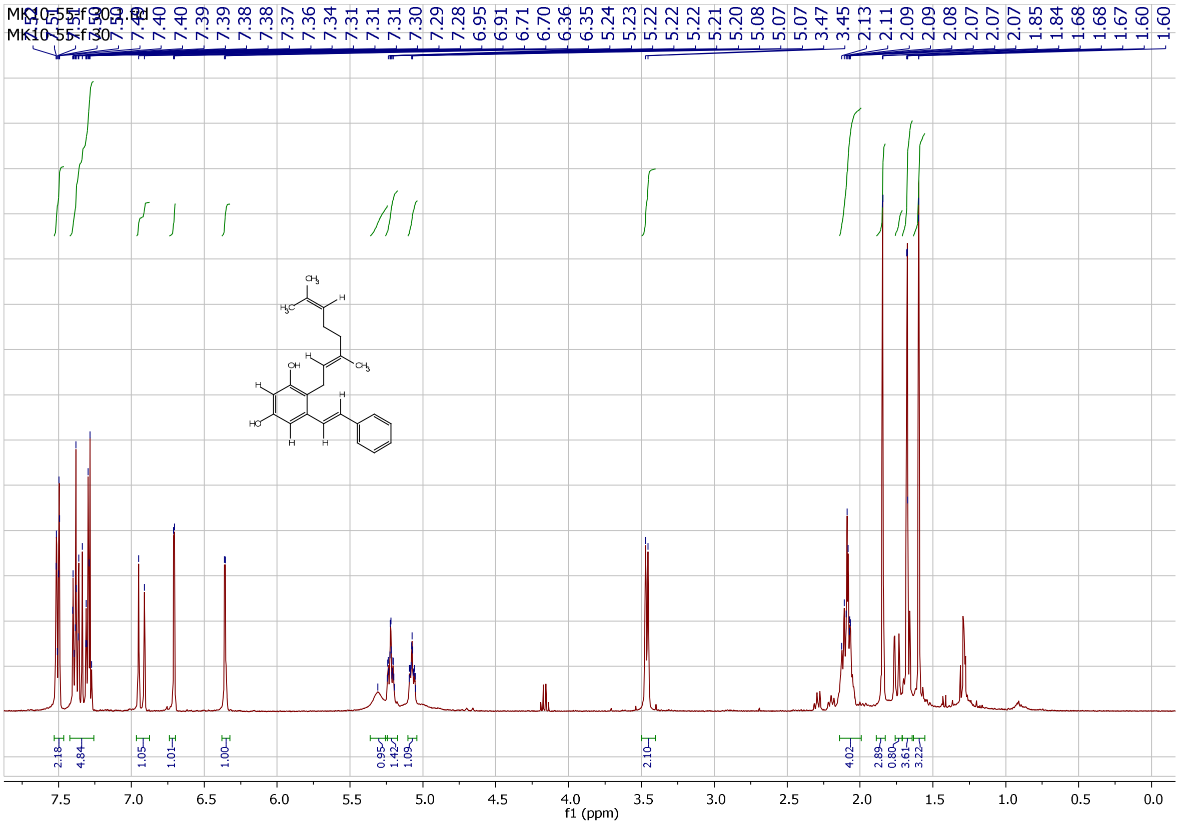

Figure S37. ^{1}H NMR (400 MHz, Chloroform-*d*) of **14.**

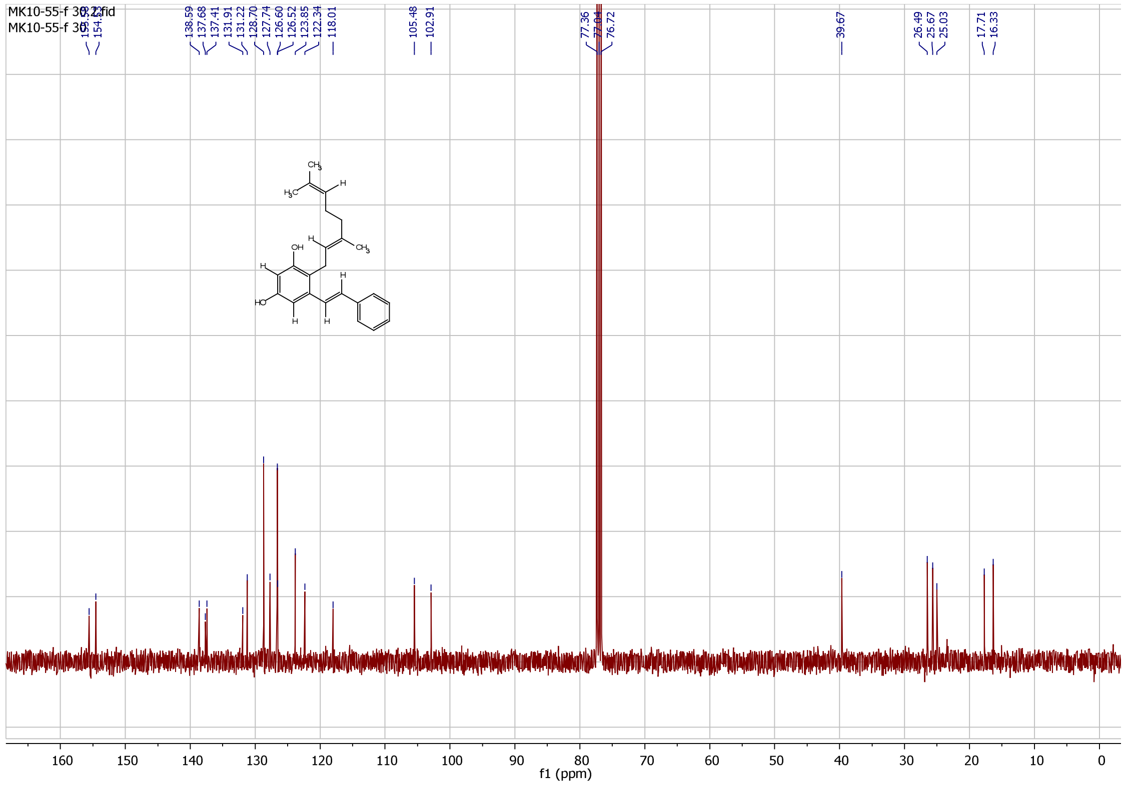

Figure S38. ^{13}C NMR (101 MHz, Chloroform-*d*) of **14.**

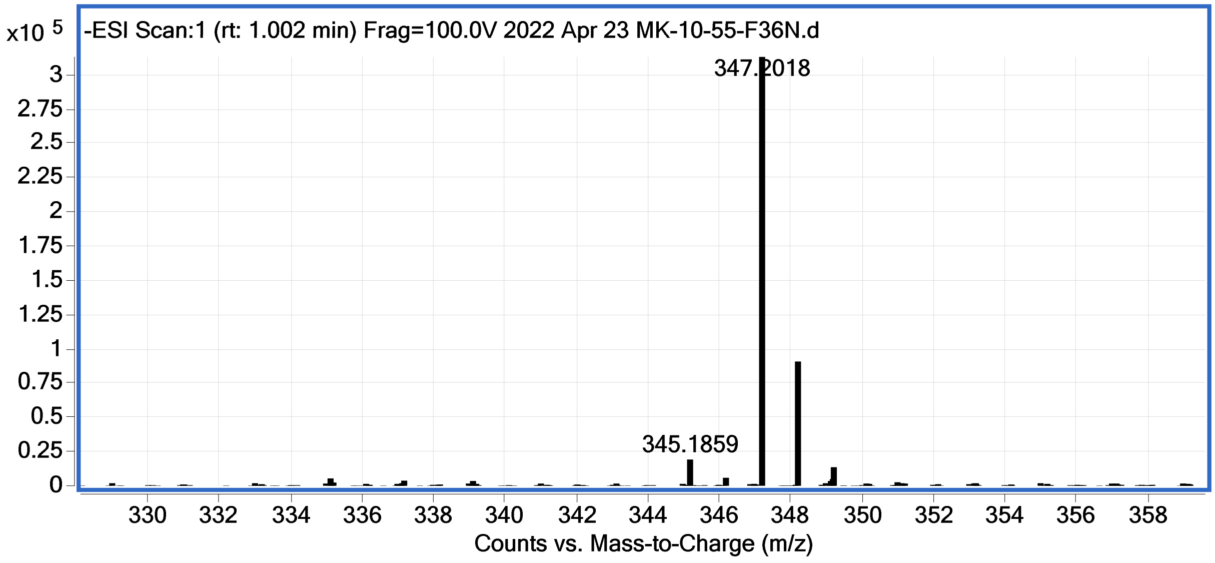

Figure S39. HRMS of 14.

Compound **15**

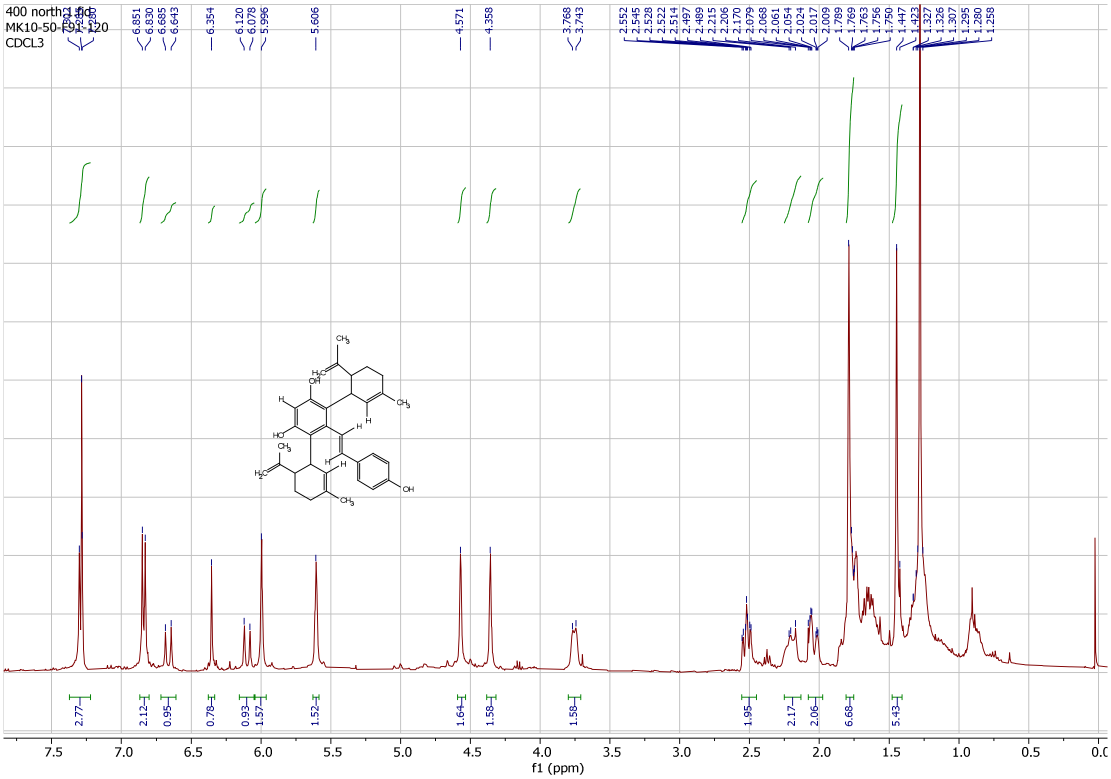

Figure S40. ^{1}H NMR (400 MHz, Chloroform-*d*) of **15.**

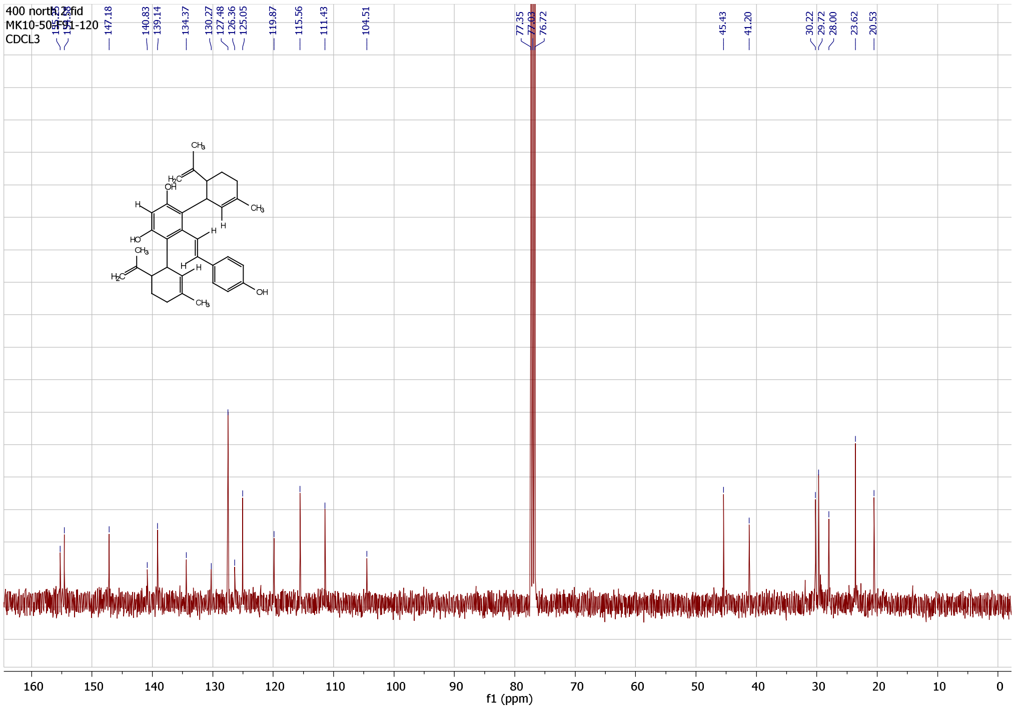

Figure S41. ^{13}C NMR (101 MHz, Chloroform-*d*) of **15.**

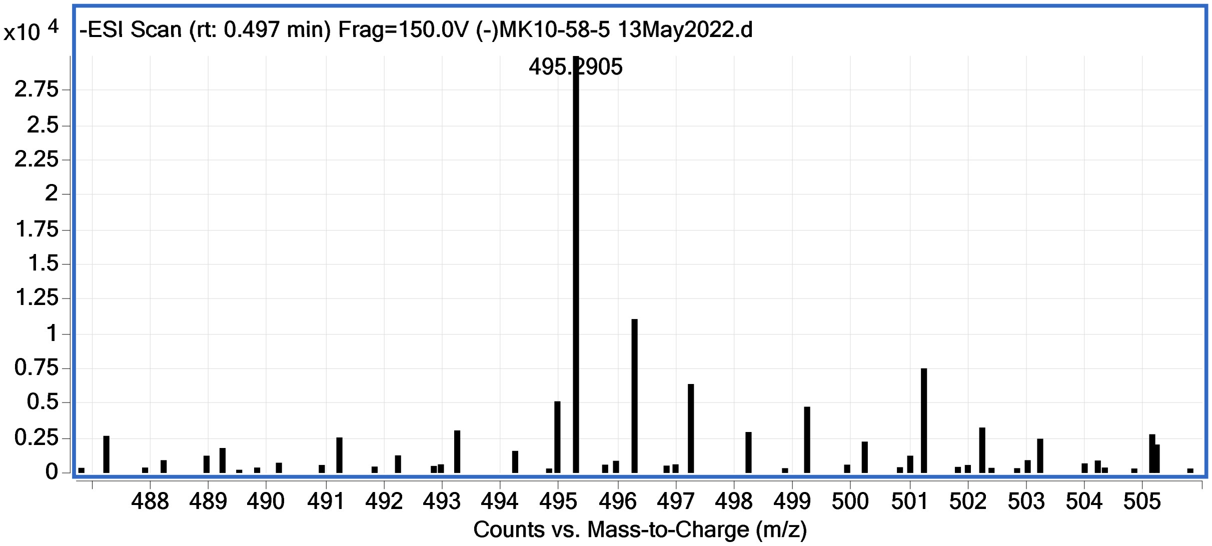

Figure S42. HRMS of 15.

Compound **16**

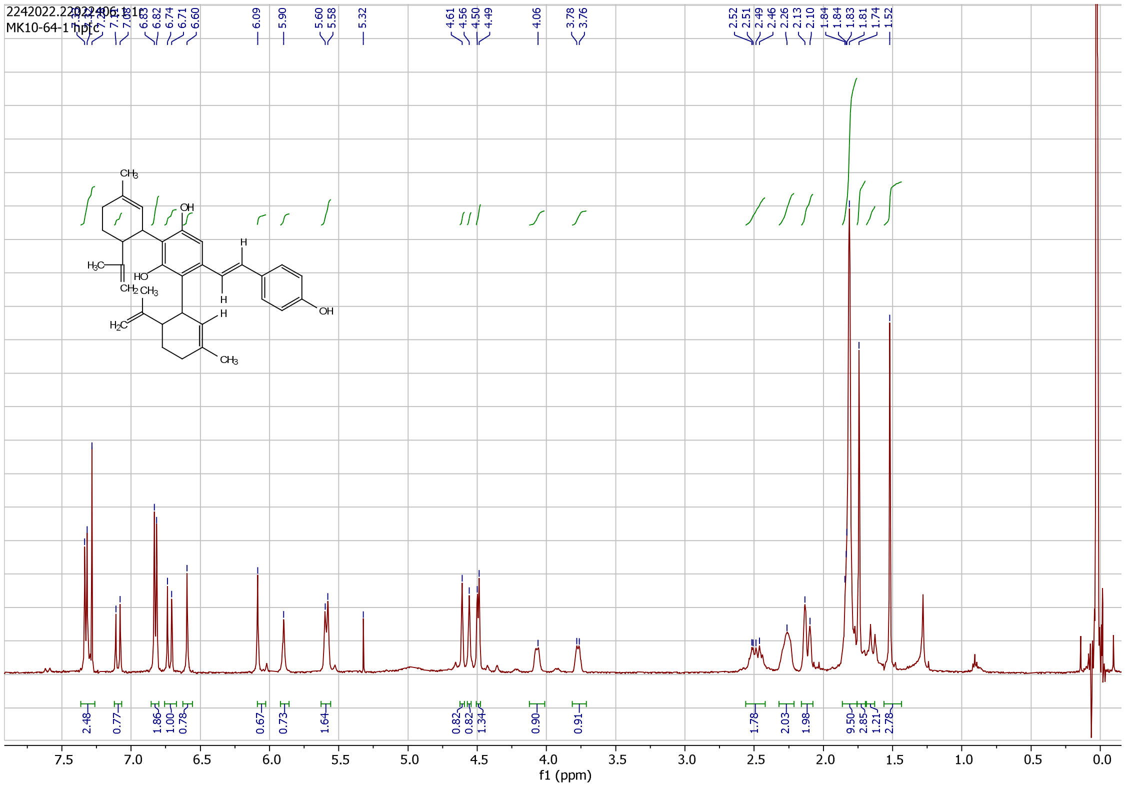

Figure S43. ^{1}H NMR (400 MHz, Chloroform-*d*) of **16.**

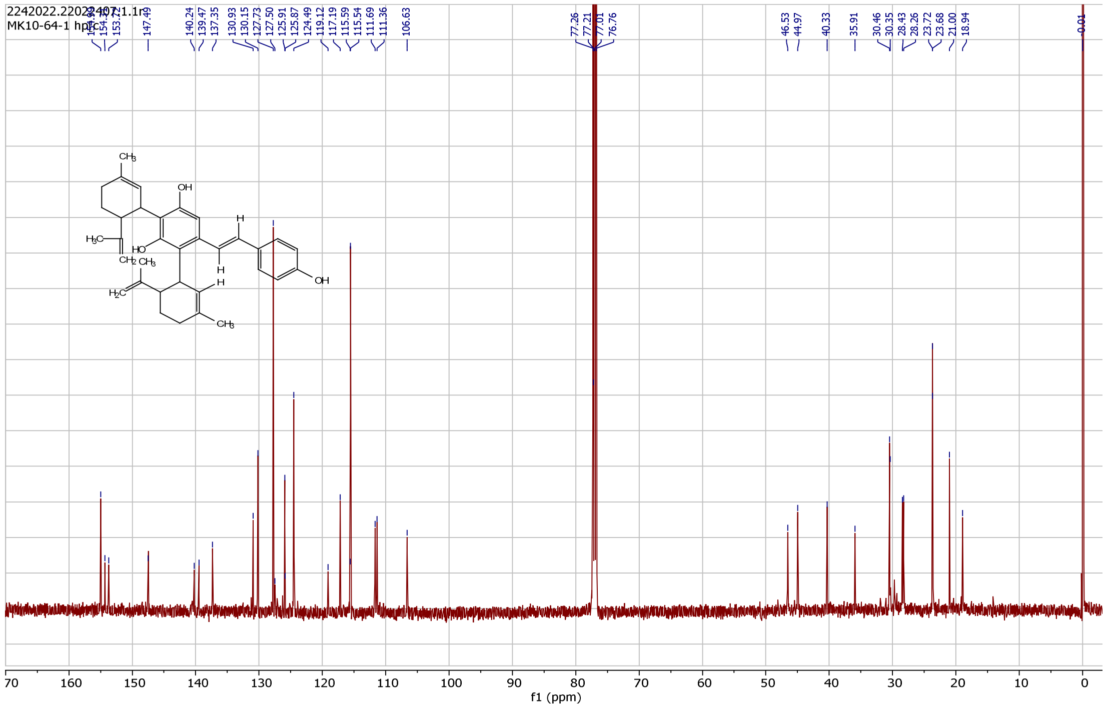

Figure S44. ^{13}C NMR (126 MHz, Chloroform-*d*) of **16.**

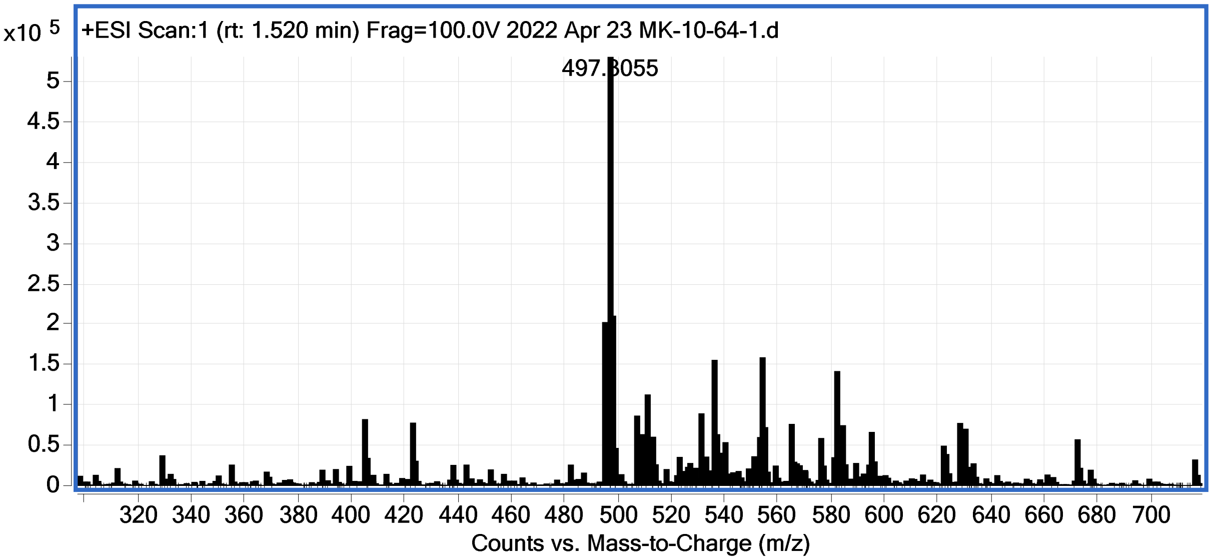

Figure S45. HRMS of 16.

Compound **17**

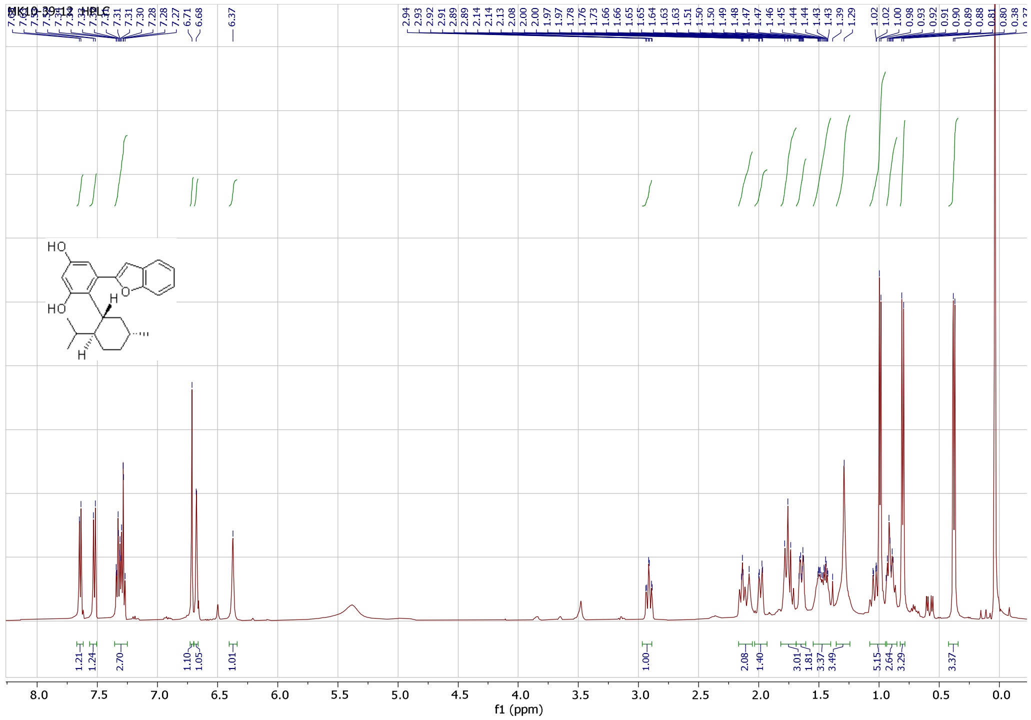

Figure S46. ^{1}H NMR (500 MHz, Chloroform-*d*) of **17.**

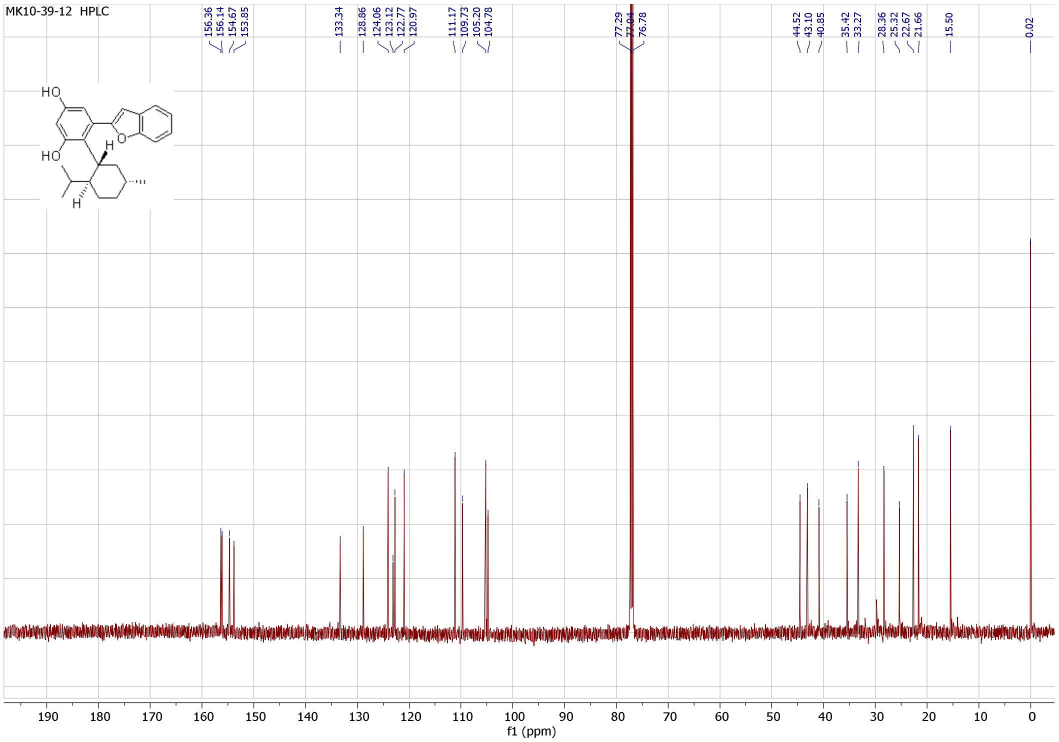

Figure S47. ^{13}C NMR (126 MHz, Chloroform-*d*) of **17.**

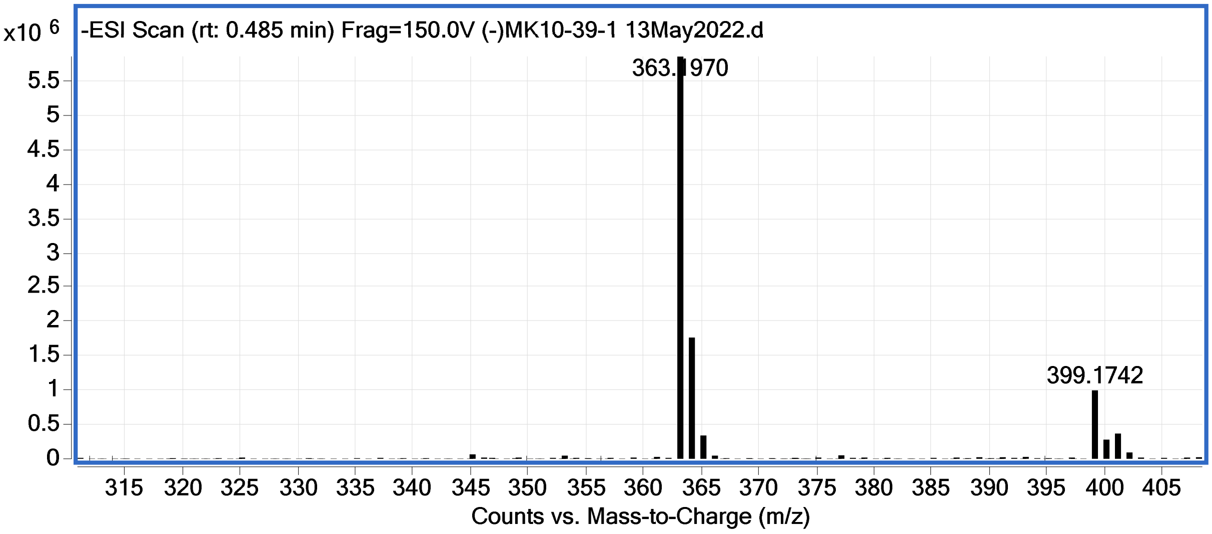

Figure S48. HRMS data of 17.

Table S1

Table S1: List of vectors used in Transfection and luciferase assays				
Pathway	Vector Name	Description	Enhancer Sequence	Source
pTK Control	TA-luc	Empty Control Reporter Vector		Affymetrix/LR0000
STAT3	pSTAT3-luc	Stat3(1) Luciferase Reporter Vector	TGCTTCCCGAATTCCCGAATTCCCGAATTCCCGAATTCCCGAATTCCCGAACGT	Affymetrix/LR0077
SMAD3/4	pSMAD-luc	Smad Luciferase Reporter Vector	AGTATGTCTAGACTGAAGTATGTCTAGACTGAAGTATGTCTAGACTGA	Affymetrix/LR0116
AP-1	P1A1-AP1-Luc	4 copies of synthetic AP-1 binding site	TGAGTCATGAGTCATGAGTCATGAGTCA	PMID: 11689104
NF-kB	pBIIX-Luc	2 copies of of binding site from immunoglobuin K promoter	GCTACAAGGGACTTTCCGCTGGGGACTTTCCAGG	PMID: 8108136
E2F	p1A1-E2F-Luc	2 copies of E2F Binding Site	TTTCGCGCTTTCGCGC	PMID: 11689104
Myc	pGL2M4-Luc	Minimal SV40 promoter-4 copies of Myc-Max binding site	CACGTGCACGTGCACGTGCACGTG	PMID: 9150134
Ets	p1A1-ETS-Luc	2 copies of inverted Ets2 binding sites	GACCGGAAGTAGTTCCGGTCGACCGGAAGTAGTTCCGGTC	PMID: 11689104
Notch	TA-CSLPromoter-Luc	2 CSL head to head binding sites upstreatm of Hes1	CAAACAAAAAATTCTTTTTCGTGAAGAACTCCAAAAAT	Signosis/ LR-2200
FOXO	pFKHR-Luc	Forkhead box 01- Forkhead Cis Element	CAAAACAACAAAACAACAAAACAACAAAACAA	Signosis/LR-2037
Wnt P	TOPFlash TCF Reporter Plasmid	Thymidine Kinase Minimal Promoter-6 Copies of HNF1 homeobox A-Luciferase	AGATCAAAGGGGGTAAGAKCAAAGGGGGTAAAATCAAAGGGGGCCCCCTTTGATCTTACCCCCTTTGATCTTACCCCCTTTGATCT	Millipore Sigma/21-170
Hedgehog	pGli1-Luc	GLI zinc finger transcription factor- 5 repeats of Gli1 binding site	GAAGACCACCCACAATGAAGACCACCCACAATGAAGACCACCCACAATGAAGACCACCCACAATGAAGACCACCCACAAT	Signosis/LR-2095
miR-21	pSicoR-mCherry-miR-21	Two Complimentary miR21 binding sites	GTACATCAACATCAGTCTGATAAGCTACCCGGGTCAACATCAGTCTGATAAGCTAG	PMID: 19419954
K-Ras	pGL4.17	From -324 to +50 relative to the start site of kRAS promoter	AGGGCGGTGTGGGAAGAGGGAAGAGGGGGAGG	PMID: 26597160
AhR	pA10CAT	From -1028 to -997 relative to CYPIA1 start site	CTCCAGGCTCTTCTCACGCAACTCCGGGGCAC	PMID: 2174107
